# Multimodal nanoparticle‐containing modified suberoylanilide hydroxamic acid polymer conjugates to mitigate immune dysfunction in severe inflammation

**DOI:** 10.1002/btm2.10611

**Published:** 2023-10-14

**Authors:** Nhu Truong, Andrea L. Cottingham, Shruti Dharmaraj, Jacob R. Shaw, Jackline Joy Martin Lasola, Christopher C. Goodis, Steven Fletcher, Ryan M. Pearson

**Affiliations:** ^1^ Department of Pharmaceutical Sciences University of Maryland School of Pharmacy Baltimore Maryland USA; ^2^ Department of Microbiology and Immunology University of Maryland School of Medicine Baltimore Maryland USA; ^3^ Marlene and Stewart Greenebaum Comprehensive Cancer Center University of Maryland School of Medicine Baltimore Maryland USA

**Keywords:** drug delivery, histone deacetylase inhibitor, inflammation, lipopolysaccharide‐induced endotoxemia, nanoparticle, sepsis

## Abstract

Excessive immune activation and immunosuppression are opposing factors that contribute to the dysregulated innate and adaptive immune responses seen in severe inflammation and sepsis. Here, a novel analog of the histone deacetylase inhibitor (HDACi), suberoylanilide hydroxamic acid (SAHA‐OH), was incorporated into immunomodulatory poly(lactic acid)‐based nanoparticles (iNP‐SAHA) by employing a prodrug approach through the covalent modification of poly(lactic‐co‐glycolic acid) (PLGA) with SAHA‐OH. iNP‐SAHA formulation allowed for controlled incorporation and delivery of SAHA‐OH from iNP‐SAHA and treatment led to multimodal biological responses including significant reductions in proinflammatory cytokine secretions and gene expression, while increasing the survival of primary macrophages under lipopolysaccharide (LPS) challenge. Using a lethal LPS‐induced endotoxemia mouse model of sepsis, iNP‐SAHA administration improved the survival of mice in a dose‐dependent manner and tended to improve survival at the lowest doses compared to iNP control. Further, iNP‐SAHA reduced the levels of plasma proinflammatory cytokines and chemokines associated with sepsis more significantly than iNP and similarly improved inflammation‐induced spleen and liver toxicity as iNP, supporting its potential polypharmacological activity. Collectively, iNP‐SAHA offers a potential drug delivery approach to modulate the multifaceted inflammatory responses observed in diseases such as sepsis.

**Translational Impact Statement**

The development of a multimodal drug delivery system that precisely incorporates a novel HDACi (SAHA‐OH) into immunomodulatory poly(lactic acid)‐based nanoparticles represents a potentially significant translational advance for the treatment of severe inflammatory diseases.

## 1 INTRODUCTION

The aberrant production of inflammatory molecules is a major contributor to the development of various immune‐mediated diseases and conditions, including sepsis.^1^ During sepsis, the innate immune system becomes activated in response to infection, which causes the overproduction and release of proinflammatory cytokines and chemokines, commonly referred to as the “cytokine storm.”^2^ These inflammatory products, if left unchecked, can lead to tissue damage, cellular and molecular dysfunction, and multi‐organ failure, ultimately culminating in death.^3^ Despite significant efforts, there is currently no FDA‐approved therapy that improves patient survival, primarily due to the complexity and profound clinical heterogeneity of the disease and the tendency of treatments that target only a single molecular pathway.^4^, ^5^ Therefore, an urgent need exists to develop effective therapeutics that target the multifaceted and dysregulated immune responses seen in sepsis.

Histone acetyltransferases (HATs) are essential enzymes involved in the epigenetic regulation of gene transcription programs by modifying chromatin histones. Activation of HATs results in increased chromatin accessibility through the transfer of an acetyl group on a ε‐amino group of a target lysine side chain within a substrate histone; conversely, histone deacetylases (HDACs) catalyze the removal of an acetyl group on lysines.^6^ Severe inflammatory conditions can cause a state of global cellular hypoacetylation due to an imbalance in HAT/HDAC activity.^7^, ^8^ To counteract this imbalance, HDAC inhibitors (HDACi) are designed to normalize acetylation profiles by reversing transcriptional silencing.^7^, ^9^, ^10^

Suberoylanilide hydroxamic acid (SAHA; vorinostat) is an FDA‐approved pan‐HDACi for cutaneous T cell lymphoma that possesses beneficial anti‐inflammatory properties, such as the ability to suppress proinflammatory cytokine production and improve survival in mouse models of sepsis.^11^, ^12^, ^13^, ^14^ The paradoxical finding that HDACis induce cancer cell apoptosis yet can induce anti‐inflammatory immune cell profiles is believed to be associated with the inherent lack of HDAC selectivity.^9^, ^15^, ^16^ A recent study from our lab discovered a beneficial modification to SAHA (SAHA‐OH), which significantly improved its concentration‐dependent toxicity profile and mitigated organ damage without compromising its anti‐inflammatory properties when compared to unmodified SAHA under lipopolysaccharide (LPS) inflammatory challenge.^17^ This improvement was attributed to a 10 to 47‐fold increase in HDAC6 selectivity as compared to HDAC 1, 2, 3, and 8. As previous preclinical interventions using HDACis to target histone modifications have been effective in sepsis, we sought to develop a drug delivery strategy to precisely control the SAHA‐OH delivery to innate immune cells for epigenetic modification of the inflammatory response.

Our lab has developed drug‐free immunomodulatory nanoparticles (iNPs) composed of poly(lactic acid) (PLA), an FDA‐approved biomaterial for use in humans with excellent biocompatibility and low immunogenicity.^18^, ^19^, ^20^, ^21^ These iNPs possess inherent immunomodulatory activity, effectively reducing the secretion of proinflammatory cytokines by innate immune cells when faced with an inflammatory challenge, such as LPS.^18^ Previous studies have shown that lactate‐mediated immune responses are dichotomous and context‐dependent,^22^, ^23^, ^24^, ^25^ despite circulating lactate levels being negatively correlated with sepsis survival.^26^, ^27^ In fact, blocking lactic acid production has been shown to improve survival in mouse models of sepsis.^28^ iNPs utilize a multimodal mechanism of action that combines physical blockade and functional reprogramming of inflammatory cell signaling to mitigate proinflammatory immune responses and improve survival in a mouse model of LPS‐induced endotoxemia.^18^, ^20^ The inherent anti‐inflammatory activity of iNPs offers an opportunity to develop a polypharmacological strategy in combination with HDACi (SAHA‐OH) to create a broad‐acting therapeutic capable of mitigating the multifaceted inflammatory responses that occur during sepsis.^29^

**FIGURE 1:**
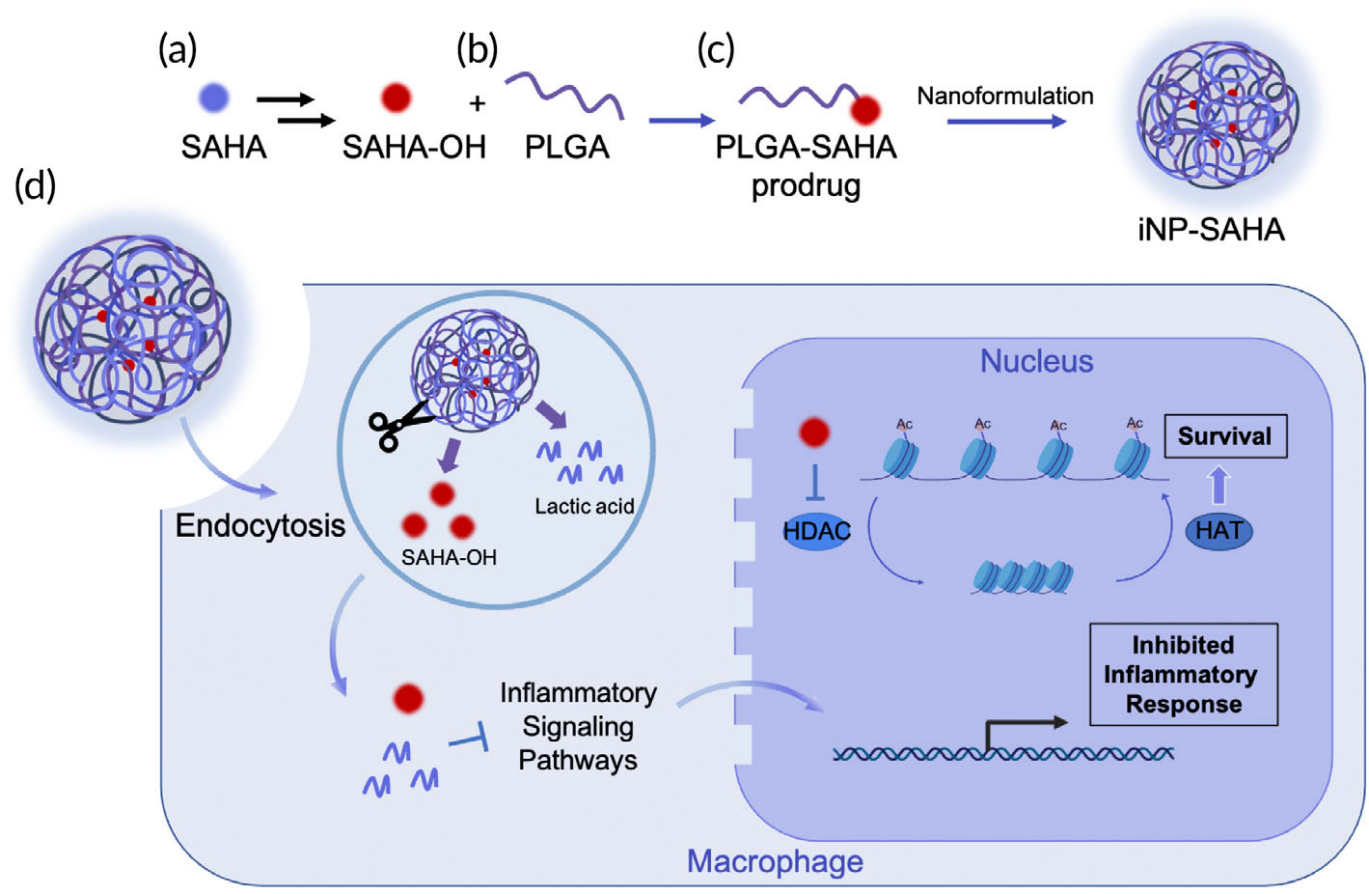
Graphical schematic of the formulation and proposed multimodal mechanism of action of iNP‐SAHA in primary macrophages. (a) SAHA was modified to SAHA‐OH and (b) precisely conjugated with PLGA to form a PLGA‐SAHA prodrug. (c) PLGA‐SAHA was used to form iNP‐SAHA nanoparticles. (d) The treatment of iNP‐SAHA to macrophages led to its internalization through endocytosis, where endosomal degradation resulted in SAHA‐OH and lactic acid as cleavage products. Both components play a role in metabolic and epigenetic reprogramming that occurs within the nucleus. Lactic acid and SAHA‐OH has demonstrated inhibitory effects on the inflammatory signaling pathway that ultimately resulted in inhibited inflammatory responses. SAHA‐OH inhibits HDACs, thus allowing HATs to transcriptionally activate gene expression for a pro‐survival response.

Here, we synthesized a poly(lactic‐co‐glycolic acid) (PLGA)‐HDACi conjugate using SAHA‐OH (PLGA‐SAHA) and formulated iNPs containing varying amounts of the conjugate (iNP‐SAHA) (Figure 1a–c) and investigated its impact on LPS‐induced inflammation using in vitro and in vivo models. We measured the ability of iNP‐SAHA treatment to induce nuclear and cytoplasmic acetylation, its effects on the production of proinflammatory cytokines induced by LPS stimulation, and cell survival using primary bone marrow‐derived macrophages (BMMØs) (Figure 1d). NanoString analysis was employed to reveal iNP‐SAHA‐mediated effects on LPS‐induced inflammatory gene expression, and the LPS‐induced endotoxemia mouse model was used to evaluate the biodistribution profile and dose‐dependent effects on survival. Lastly, the effect of iNP‐SAHA treatment on the modulation of systemic cytokines and organ toxicity was evaluated. Overall, the combinatorial anti‐inflammatory and pro‐survival effects provided by iNP‐SAHA provides a potential approach to attenuate systemic acute immune activation seen in sepsis. We anticipate that this multimodal, polypharmacological drug delivery approach has the potential to overcome the dysregulated immune responses observed in severe inflammation and sepsis to improve patient outcomes.

## 2 MATERIALS AND METHODS

### 2.1 Materials

Acid‐terminated poly(D,L‐lactide) (PLA) of low inherent viscosity in hexafluoro‐2‐propanol ~0.21 dL/g (approximate MW 11,300 g/mol) and acid‐terminated poly(D,L‐lactide‐co‐glycolide) (PLGA), of low inherent viscosity in hexafuoro‐2‐propanol ~0.17 dL/g (approximate MW 4200 g/mol) were purchased from Lactel Absorbable Polymers located at Birmingham, AL. Poly(ethylene‐alt‐maleic anhydride) (PEMA) (MW 400,000 g/mol) was purchased from Polysciences, Inc. located at Warrington, PA. LPS (*Escherichia coli* O111:B4, #L2630) and RPMI 1640 (supplemented with L‐glutamine, #R8758) were obtained from Millipore Sigma (St. Louis, MO). Penicillin–streptomycin, Versene, and NuPAGE™ 12% Bis‐Tris 1.0 mm Mini Protein Gel (#NPO343BOX) were purchased from Thermo Fisher Scientific (Waltham, MA). Fetal bovine serum (FBS, #97068‐085) was purchased from VWR (Radnor, PA). L929 fibroblast cells (NCTC clone 929) were purchased from the American Type Culture Collection (ATCC, Manassas, VA). 2× SDS/PAGE sample buffer was produced using 4% SDS, 5.7 M β‐mercaptoethanol, 0.2 M Tris/HCl, pH 6.8, 20% glycerol, and 5 mM EDTA. All chemicals were of analytical grade and obtained from Millipore Sigma located in St. Louis, MO, and CombiBlocks located in San Diego, CA.

### 2.2 PLGA‐SAHA conjugation reaction

**FIGURE 2:**
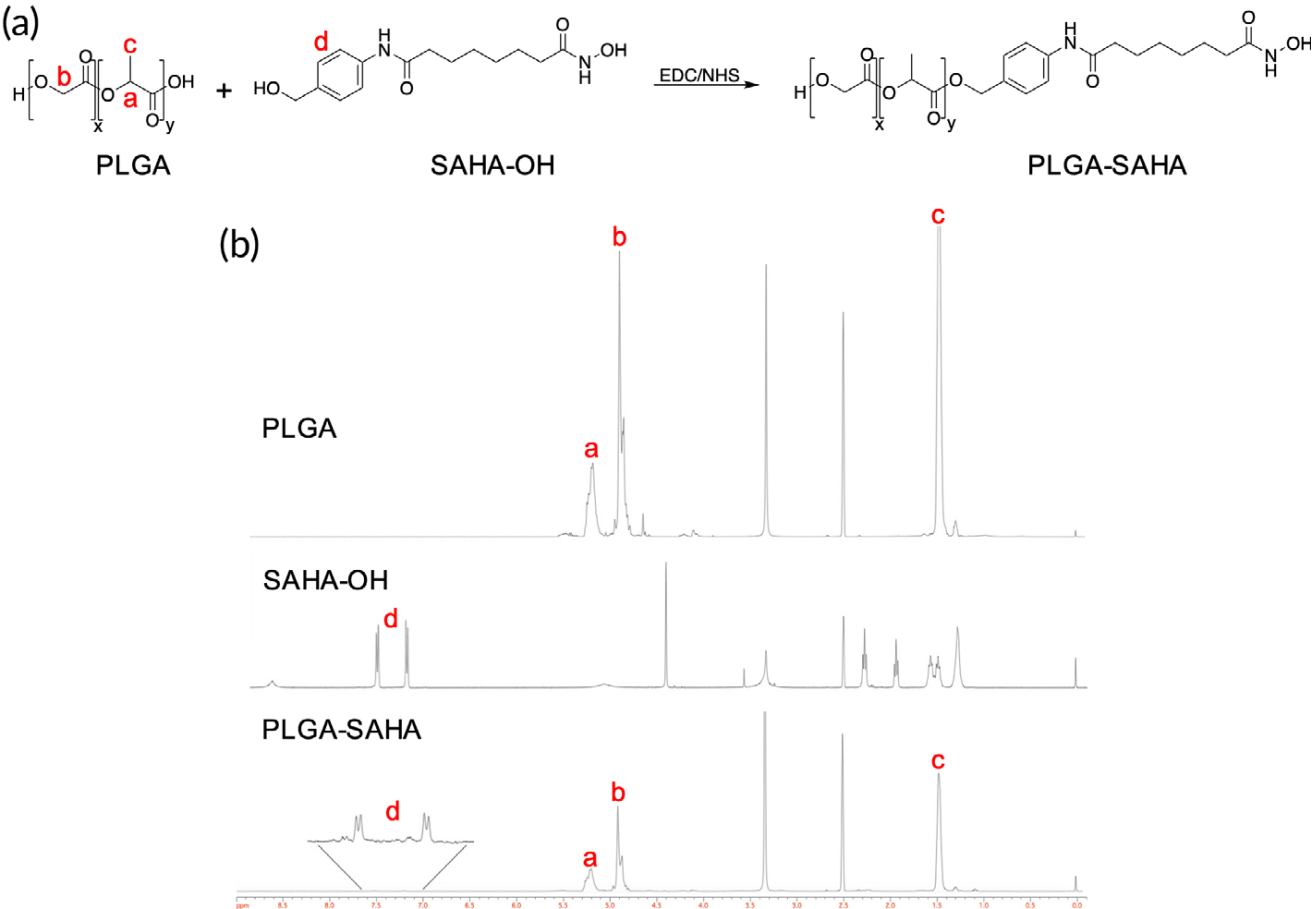
Synthesis and characterization of PLGA‐SAHA conjugates. (a) EDC/NHS reaction of PLGA and SAHA‐OH to synthesize PLGA‐SAHA conjugates. (b) ^1^H‐NMR spectrum of PLGA, SAHA‐OH, and PLGA‐SAHA measured in DMSO‐d_6_ (calibrated at 2.5 ppm). Coupling efficiency was measured to be 77.5%. ^1^H NMR (400 MHz, DMSO‐d_6_) of PLGA: δ (ppm) 5.20 (m, 1H), 4.91 (s, 2H), 1.47 (m, 3H). ^1^H NMR (400 MHz, DMSO‐d_6_) of SAHA‐OH: δ (ppm) 7.52 (d, J = 8 Hz, 2H), 7.19 (d, J = 8.8 Hz, 2H), 4.40 (s, 2H), 2.26 (t, J = 7.2 Hz, 2H), 1.92 (t, J = 6.8 Hz, 2H), 1.55 (t, J = 6.4 Hz, 2H), 1.47 (t, J = 6.8 Hz, 2H). ^1^H NMR (400 MHz, DMSO‐d_6_) of PLGA‐SAHA: δ (ppm) 7.52 (d, J = 8 Hz, 2H), 7.19 (d, J = 8.8 Hz, 2H), 5.20 (m, 1H), 4.91 (s, 2H), 4.40 (s, 2H), 2.26 (t, J = 7.2 Hz, 2H), 1.92 (t, J = 6.8 Hz, 2H), 1.55 (t, J = 6.4 Hz, 2H), 1.47 (m, 2H).

SAHA‐OH was synthesized following a previously described method.^17^ SAHA‐OH was conjugated to acid‐terminated PLGA by an EDC/NHS carbodiimide chemistry reaction (Figure 2a). First, PLGA was dissolved at 20 mg/mL in DMSO and magnetically stirred at 25°C. SAHA‐OH was dissolved at 10 mg/mL in DMSO. Triethylamine (TEA) (Millipore Sigma, St. Louis, MO) was added at 5× excess molar ratio to SAHA‐OH. N‐(3‐dimethylaminopropyl)‐N′‐ethylcarbodiimide hydrochloride (EDC) (Millipore Sigma, St. Louis, MO) crosslinker was dissolved at 20 mg/mL in DMSO and added dropwise to the stirring PLGA solution for 5 min. N‐hydroxysuccinimide (NHS) (Thermo Fisher Scientific, Waltham, MA) was dissolved at 5 mg/mL in DMSO and added dropwise to the EDC‐activated PLGA solution and stirred for 10 min. SAHA‐OH with TEA was added dropwise to the EDC/NHS‐activated PLGA solution and stirred overnight to allow the reaction to progress. The resulting PLGA‐SAHA conjugate was purified through dialysis utilizing a 3500 molecular weight cut‐off membrane (Thermo Fisher Scientific, Waltham, MA). The conjugate was first dialyzed against two DMSO exchanges (500 mL) over a course of 6 h. To exchange the DMSO solvent to water, a series of six distilled water exchanges (4 L) over 2 days occurred. The conjugates were then frozen at −80°C for at least 2 h prior to lyophilization using a Freezone 4.5 L −50°C Complete Freeze Dryer System (Labconco, Missouri, USA) for 2 days. The coupling efficiency was calculated through ^1^H NMR. ^1^H NMR (400 MHz, DMSO‐d_6_) of PLGA: δ (ppm) 5.20 (m, 1H), 4.91 (s, 2H), 1.47 (m, 3H). ^1^H NMR (400 MHz, DMSO‐d_6_) of SAHA‐OH: δ (ppm) 7.52 (d, J = 8 Hz, 2H), 7.19 (d, J = 8.8 Hz, 2H), 4.40 (s, 2H), 2.26 (t, J = 7.2 Hz, 2H), 1.92 (t, J = 6.8 Hz, 2H), 1.55 (t, J = 6.4 Hz, 2H), 1.47 (t, J = 6.8 Hz, 2H). ^1^H NMR (400 MHz, DMSO‐d_6_) of PLGA‐SAHA: δ (ppm) 7.52 (d, J = 8 Hz, 2H), 7.19 (d, J = 8.8 Hz, 2H), 5.20 (m, 1H), 4.91 (s, 2H), 4.40 (s, 2H), 2.26 (t, J = 7.2 Hz, 2H), 1.92 (t, J = 6.8 Hz, 2H), 1.55 (t, J = 6.4 Hz, 2H), 1.47 (m, 2H).

### 2.3 Preparation and characterization of nanoparticles

iNP, iNP‐SAHA, iNP‐Cy5.5, and iNP‐SAHA‐Cy5.5 were prepared by the single oil‐in‐water (o/w) emulsion solvent evaporation technique using a similar method as previously described.^18^, ^21^ Briefly, PLA was dissolved in ethyl acetate at 80 mg/mL to generate iNP. For iNP‐SAHA, SAHA‐OH loading was determined from the coupling efficiency using ^1^H NMR. Pre‐determined amounts of PLGA‐SAHA were added to PLA at 50 mg/mL in ethyl acetate to formulate two loadings of SAHA‐OH into iNPs: 9.8 μg/mg iNP‐SAHA_Low_, and 62.3 μg/mg iNP‐SAHA_High_. Cyanine 5.5 (Lumiprobe, Cockeysville, MD) was first conjugated to PLGA (PLGA‐Cy5.5) using EDC/NHS carbodiimide chemistry, similarly to the PLGA‐SAHA reaction. PLGA‐Cy5.5 was added at 0.5% (w/w) to PLA at 50 mg/mL. iNP, iNP‐SAHA, iNP‐Cy5.5, and iNP‐SAHA‐Cy5.5 formulation was similarly performed, where 20 mL of 1% PEMA was added and sonicated for 30 s at 100% amplitude using a Cole‐Parmer 500‐Watt Ultrasonic Homogenizer. The resulting o/w emulsion was then added to magnetically stirred 0.5% PEMA overnight until all the ethyl acetate evaporated. The nanoparticles were then collected by centrifugation at 12,000×*g* for 20 min at 4°C and washed with 40 mL of MilliQ water, for a total of four washes. Cryoprotectant (4% w/v sucrose, 3% w/v mannitol) was added to the nanoparticle suspension, frozen at −80°C, and lyophilized for 2 days. The size, zeta potential, and polydispersity index (PDI) of the nanoparticles were determined by dynamic light scattering (DLS) using a Malvern Zetasizer Nano ZSP (Malvern Instruments Inc., Westborough, MA) as previously described.^21^, ^30^ For these measurements, iNPs were dispersed in water (pH 6) at room temperature. Lyophilized iNP, iNP‐SAHA_Low_, and iNP‐SAHA_High_ were adhered to an aluminum stub and sputter‐coated with platinum and palladium at 20 mA for 20 s for scanning electron microscopy (SEM) analysis.^21^ A FEI Quanta 200 (FEI, Hillsboro, OR) SEM was used to capture the images at an accelerating voltage of 15 kV at 12.3 mm working distance using a magnification of 34,000×. The cumulative release studies were performed using iNP‐SAHA_Low_ and iNP‐SAHA_High_ examined in pH 5 acetate buffer and pH 7.4 PBS buffer. Samples were collected at specific timed intervals over the course of 14 days at 37°C on a rotating shaker. The quantification of SAHA was performed using a diazocoupling method using sulfanilic acid and α‐naphthylamine and measured on a SpectraMax plate reader (Molecular Devices, San Jose, CA), as previously described.^31^

### 2.4 Mice

All procedures and experiments involving mice were performed in compliance with the protocols established by the University of Maryland, Baltimore Institutional Animal Care and Use Committee (IACUC) (Protocol #0721010) as well as the ARRIVE guidelines. Five‐ to 7‐week‐old C57BL/6J (male and female) were purchased from The Jackson Laboratories (Bar Harbor, ME). The mice were kept in a facility at the University of Maryland, Baltimore Veterinary Resources, which maintained specific pathogen‐free conditions. Male C57BL/6 mice were selected for all LPS‐induced endotoxemia studies to eliminate potential hormone‐related influences on the data.^32^

### 2.5 Bone marrow‐derived macrophages

The generation of BMMØs from the bone marrow of C57BL/6 mice followed previously published methods.^33^ As established, the femurs and tibias from male or female C57BL/6J mice were isolated and flushed with complete BMMØ media (RPMI 1640 supplemented with L‐glutamine, penicillin (100 units/mL), streptomycin (100 μg/mL), 10% heat‐inactivated FBS, and 20% L929 cell‐conditioned media). The bone marrow cells were filtered and plated in uncoated 10 cm petri dishes. The media was replaced every 3 days on Days 0, 3, 6, and 8 and incubated at 37°C at 5% CO_2_. Days 8–10 BMMØs were lifted using Versene to be used for subsequent experiments. Trypan blue solution determined the cell number and viability using the EVE™ Automated Cell Counter (NanoEntek, Waltham, MA).

### 2.6 Western blot

In sterile 6‐well plates, Day 8 BMMØs were seeded at 1 × 10^6^ cells/well and incubated at 37°C and 5% CO_2_ overnight to allow for cell adherence, as previously described.^17^ BMMØs were treated with 300 μg/mL iNP, 300 μg/mL iNP‐SAHA_Low_, or 300 μg/mL iNP‐SAHA_High_ and incubated for 4, 9, 27, or 48 h. Cells were isolated using RIPA Buffer (#R0278) (Millipore Sigma, St. Louis, MO) containing Halt™ Protease Inhibitor Cocktail (#78429) (Thermo Fisher, Waltham MA) and scraped using a cell scraper (VWR, Radnor, PA). A 1/8″ tip using a Cole‐Parmer 500‐Watt Ultrasonic Homogenizer at 40% amplitude for 10 s on ice was used to sonicate the cell lysates, then centrifuged at 4°C for 20 min at 12,000×*g*. The supernatant was extracted and frozen at −80°C. A 50/50 sample to 2× SDS/PAGE sample buffer produced protein lysates. SDS/PAGE was used to separate proteins then immunoblotted using Histone H3 (D1H2) (#4499) Rabbit mAb, Acetyl‐Histone H3 (Lys9/Lys14) (#9677) Rabbit mAb, α‐Tubulin (11H10) (#2125) Rabbit mAb, Acetyl‐α‐Tubulin (Lys40) (D20G3) (#5335) Rabbit mAb, and β‐Actin (D6A8) (#8457) primary antibodies (Cell Signaling Technology, Danvers, MA). Enhanced luminol‐based chemiluminescent (ECL) was used for detection of the western blot. Quantification of bands was performed using Image J.

### 2.7 Immunocytochemistry staining of BMMØs

To examine the uptake of iNP‐SAHA, Day 8 BMMØs were seeded at 0.5 × 10^5^ cells/well in complete BMMØ media in sterile eight‐well chamber slides incubated at 37°C and 5% CO_2_ overnight to allow for cell adherence. The following day, media was replaced with fresh complete media supplemented with 300 μg/mL of iNP‐SAHA_Low_‐Cy5.5 or iNP‐SAHA_High_‐Cy5.5 formulations. Three hours later, excess iNP‐SAHA‐Cy5.5 was removed by washing twice with PBS followed by replacing with complete RPMI 1640 medium containing 100 ng/mL LPS. After 48 h, cells were fixed with fixation buffer (BioLegend, San Diego, CA) and blocked with blocking buffer (CST, Danvers, MA) (#12411) per manufacturer's instructions. Cells were then stained with rabbit acetyl‐histone H3 (K9K14) (#9677S) overnight at 4°C. The next day, cells were rinsed with PBS and stained with goat anti‐rabbit IgG (H + L), F(ab′)2 Fragment (Alexa Fluor® 488 Conjugate) (#4412) for 1 h. NucBlue Live ReadyProbes Reagent (Hoechst 33342) (#R37605) was then added to each well and incubated for 15 min. Fluoromount‐G Mounting Medium with DAPI (#00‐4959‐52) was added to the top of the chamber slides to seal the coverslip and cured overnight at room temperature. The cells were then imaged using the Nikon Eclipse Ti‐2 confocal microscope (Tokyo, Japan) within 2 days. Quantification of fluorescence was performed using Image J.

### 2.8 NanoString nCounter gene expression assay

Day 8 BMMØs were seeded at 3 × 10^6^ cells/well in sterile six‐well plates incubated at 37°C and 5% CO_2_ overnight to allow for cell adherence. BMMØs were treated with 300 μg/mL iNP or 300 μg/mL iNP‐SAHA_High_ and incubated for 3 h. Washing with PBS twice allowed for the removal of excess NPs followed by replacing with complete RPMI 1640 medium containing 100 ng/mL LPS. Three hours later, cells were collected and isolated for their RNA using the RNeasy Mini Kit following manufacturer's instructions (Qiagen, Hilden, Germany). The purified RNA was quantified via Nanodrop One (Fisher Scientific, Hampton, NH) and samples were sent to the Institute for Genome Sciences (IGS) Genomics Resource Center (University of Maryland, Baltimore, MD). Nanostring analysis was performed by the IGS core using the nCounter XT CodeSet [XT_PGX_MmV2_Inflammation] (#115000082) (NanoString Technologies, Seattle, WA).

### 2.9 Cytokine secretion of BMMØs following LPS challenge

In sterile 24‐well plates, Day 8 BMMØs were seeded at 1 × 10^5^ cells/well in complete BMMØ media incubated at 37°C and 5% CO_2_ overnight, as previously described.^17^ Subsequently, the media was replaced with BMMØ complete media supplemented with 30 μM SAHA‐OH, 300 μg/mL of iNP, 300 μg/mL iNP‐SAHA_Low_, or 300 μg/mL iNP‐SAHA_High_. Three hours later, PBS washing allowed for the removal of excess iNP or iNP‐SAHA followed by replacing with complete BMMØ media containing 100 ng/mL LPS. The cell culture supernatants were collected 48 h later and analyzed by MAGPIX Luminex bead‐based multiplex ELISA (Thermo Fisher Scientific, Waltham, MA) for seven various chemokines and cytokines (IFNβ, IL‐10, IL‐6, IL‐1β, MCP‐1 [CCL2], TNFα, and GROα [CXCL1]), and data were analyzed using the Luminex xPONENT software (Millipore).

### 2.10 FITC annexin V and propidium iodide staining for detection of apoptosis

In sterile 24‐well plates, Day 8 BMMØs were seeded at 1 × 10^5^ cells/well in complete BMMØ media incubated at 37°C and 5% CO_2_ overnight, as previously described.^17^ Subsequently, the media was replaced with BMMØ complete media supplemented with 30 μM SAHA‐OH, 300 μg/mL iNP, 300 μg/mL iNP‐SAHA_Low_, or 300 μg/mL iNP‐SAHA_High_. Three hours later, PBS washing allowed for the removal of excess iNP or iNP‐SAHA followed by replacing with complete BMMØ media containing 100 ng/mL LPS. The cells were lifted using Versene after 48 h and washed with MACS buffer (cold PBS supplemented with 1% FBS and 0.4% 0.5 M EDTA [Quality Biological, Gaithersburg, MD]). FcR blocking (purified anti‐mouse CD16/32 Ab [#101301] [BioLegend, San Diego, CA]) was performed prior to FITC‐annexin V (AV) and propidium iodide (PI) staining [#640914) (BioLegend, San Diego, CA) for apoptosis and cell death, according to the manufacturer's instructions. Becton Dickinson LSR II or Becton Dickinson Canto II flow cytometer was used to analyze the flow cytometry samples. FCS Express 7 Flow Cytometry De Novo Software (De Novo, Glendale, CA) was used for flow data processing.

### 2.11 Biodistribution

Male C57BL/6 mice (5–7 weeks) were randomly divided into three groups, and subjected to intraperitoneal (i.p.) injections of either saline, 0.01 mg/mouse PLGA‐Cy5.5 conjugates, or 2 mg/mouse iNP‐Cy5.5 for 3 h. Subsequently, mice were subjected to either saline or 20 mg/kg LPS challenge for 3 h. Various organs were isolated (spleen, kidneys, liver, heart, lung, and GI tract [stomach, pancreas, cecum, and large and small intestines]) and analyzed on the Xenogen in vivo imaging system (IVIS) Spectrum Optical (PerkinElmer, Waltham, MA). Excitation and emission used were 675 and 720 nm, respectively.

### 2.12 Survival

LPS endotoxemia was induced using male C57BL/6J mice (5–7 weeks) that were randomly divided into various groups and subjected to either i.p. injection with 0.5, 1 mg, or 2 mg of iNP or iNP‐SAHA or 50 mg/kg SAHA‐OH 3 h prior to i.p. LPS injection (30 mg/kg). Mice were monitored for a period of 7 days compliant to the protocols set forth by the University of Maryland Animal Care and Use Committee. Mice noted by acute loss of function and non‐sensitivity to touch were euthanized immediately at a humane endpoint.

### 2.13 Plasma cytokine secretions in the LPS‐induced endotoxemia mouse model

Male C57BL/6J mice (5–7 weeks) were randomly divided into three groups, then subjected to i.p. injection with 2 mg of iNP, 2 mg iNP‐SAHA, or saline 3 h prior to i.p. injection of 20 mg/kg LPS. Cardiac blood draws were performed 3 h post‐LPS injections, as previously described.^17^ Within 30 min of collection, the blood was centrifuged at 1000×*g* for 10 min. The plasma was extracted and processed using the MAGPIX Luminex bead‐based multiplex ELISA (Thermo Fisher Scientific, Waltham, MA) diluted 100‐fold and measured for 26 various cytokines and chemokines, and data were analyzed using the Luminex xPONENT software (Millipore) as per manufacturer's instructions. The 26‐plex panel include murine IL‐1β, IL‐2, IL‐4, IL‐5, IL‐6, IL‐9, IL‐10, IL‐12p70, IL‐13, IL‐17A (CTLA‐8), IL‐18, IL‐22, IL‐23, IL‐27, GM‐CSF, IFNγ, TNFα, MCP‐1 (CCL2), MIP‐1α (CCL3), MIP‐1β (CCL4), RANTES (CCL5), MCP‐3 (CCL7), Eotaxin (CCL11), GROα (CXCL1), MIP‐2α (CXCL2), and IP‐10 (CXCL10).

### 2.14 Hematoxylin and eosin histological sectioning

Male C57BL/6J mice (5–7 weeks) were randomly divided into four groups, then subjected to i.p. injection with 2 mg of iNP, 2 mg iNP‐SAHA, or saline 3 h prior to i.p. injection of 20 mg/kg LPS. No treatment (NT) mice did not receive LPS challenge. Whole body perfusion was performed 3 h post‐LPS challenge by slowly flushing 10 mL of 37°C PBS through the heart, as previously described.^17^ The liver and spleen were isolated, fixed in 10% buffered formalin, and paraffin embedded and sectioned into 5 μm slices. Hematoxylin and eosin (H&E) staining defined histological tissue architecture using standard procedures by the Pathology Biorepository Shared Services Core at University of Maryland, Baltimore.^34^, ^35^

### 2.15 Statistical analysis

Data statistical analyses were performed using GraphPad Prism 9 (San Diego, CA). All data was plotted as mean ± SD. The significant differences were determined by one‐way ANOVA along with Tukey's multiple comparison test. Kaplan–Meier survival curve and statistical significance of mouse survival were determined with a log‐rank (Mantel‐Cox) *χ* ^2^ test. *p* < 0.05 was considered statistically significant. Determination of mice sample size for in vivo experiments was determined using a priori power analysis at 80% power and *p* = 0.05 through the G*Power software.

## 3 RESULTS

### 3.1 PLGA‐SAHA conjugate synthesis and characterization

We previously synthesized and characterized SAHA‐OH for its potent anti‐inflammatory properties and reduced toxicity compared to its parent compound, SAHA (Figure 1a).^17^ To generate the pro‐drug PLGA‐SAHA conjugates, we conjugated SAHA‐OH to carboxyl‐terminated PLGA using EDC/NHS chemistry (Figures 1b and 2a). PLGA‐SAHA conjugates were purified using dialysis first against DMSO, then followed by MilliQ H_2_O with multiple solvent exchanges. The coupling of SAHA‐OH to PLGA was confirmed using ^1^H‐NMR, which showed a 77.5% coupling efficiency (Figure 2b).

### 3.2 Formulation of iNP‐SAHA and its ability to deliver active SAHA‐OH to BMMØs for histone modification

**FIGURE 3:**
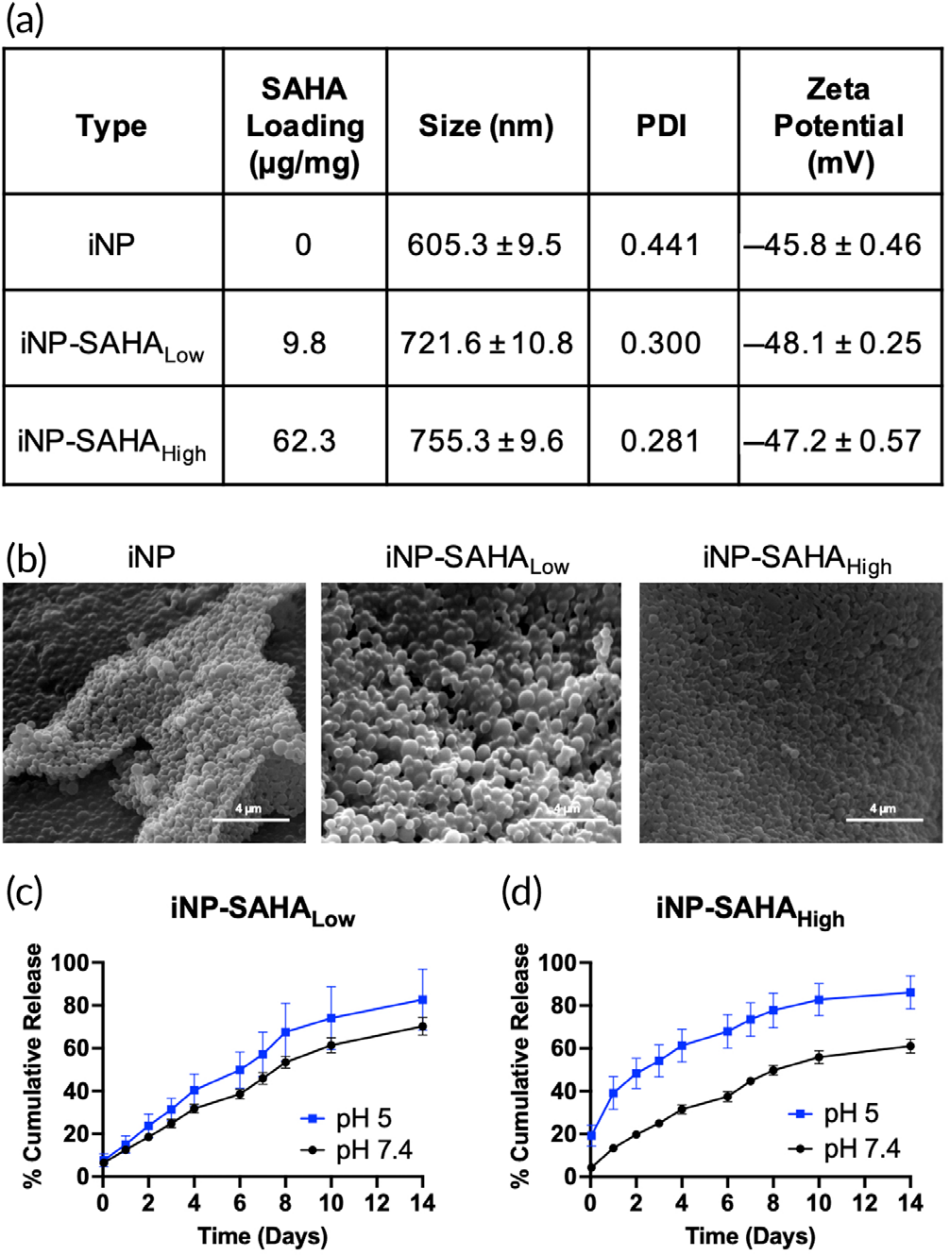
Characterization of iNP and iNP‐SAHA. (a) DLS characterization determined the particle size (nm), polydispersity index (PDI), and zeta potential (mV). iNP = drug‐free immunomodulatory nanoparticles containing no SAHA. Two loadings of iNP‐SAHA were formulated and labeled as Low and High. (b) Scanning electron microscopy (SEM) images of iNPs, iNP‐SAHA_Low_, and iNP‐SAHA_High_. The images were visualized at an accelerating voltage of 15 kV and a working distance of 12.3 mm. Magnification 34,000× was used. Scale bar is 4 μm. (c, d) Cumulative SAHA drug release studies were performed over 14 days on (c) iNP‐SAHA_Low_ and (d) iNP‐SAHA_High_ in pH 5 acetate buffer and pH 7.4 PBS buffer. All data is expressed as means ± SD (*n* = 3).

We next prepared two types of iNP‐SAHA with different loadings by mixing PLGA‐SAHA conjugate with unmodified PLA polymer at precise stoichiometric ratios followed by formulation using a single emulsion‐solvent evaporation technique (i.e., iNP‐SAHA_Low_ containing 9.8 μg SAHA‐OH/mg and iNP‐SAHA_High_ containing 62.3 μg SAHA‐OH/mg) (Figure S1). As shown in Figure 3a, iNP‐SAHA and drug‐free iNP control formulations were approximately 600–760 nm in size with highly negative zeta potentials (<−40 mV), properties that are favorable for delivery to phagocytic immune cells.^18^, ^36^ A corresponding set of fluorescently labeled iNP‐Cy5.5 and iNP‐SAHA‐Cy5.5 were also prepared using similar methods, as previously described (Table S1).^20^ The addition of SAHA‐OH to iNPs (iNP‐SAHA_Low_ and iNP‐SAHA_High_) did not affect the spherical morphology during iNP formulation, as shown through SEM analysis (Figure 3b). Additionally, the cumulative release of SAHA from iNP‐SAHA_Low_ and iNP‐SAHA_High_ performed similarly over the course of 14 days (Figure 3c,d). We observed slightly higher SAHA release in pH 5 acetate buffer (~80%) as compared to pH 7.4 PBS buffer (~60%–70%).

**FIGURE 4:**
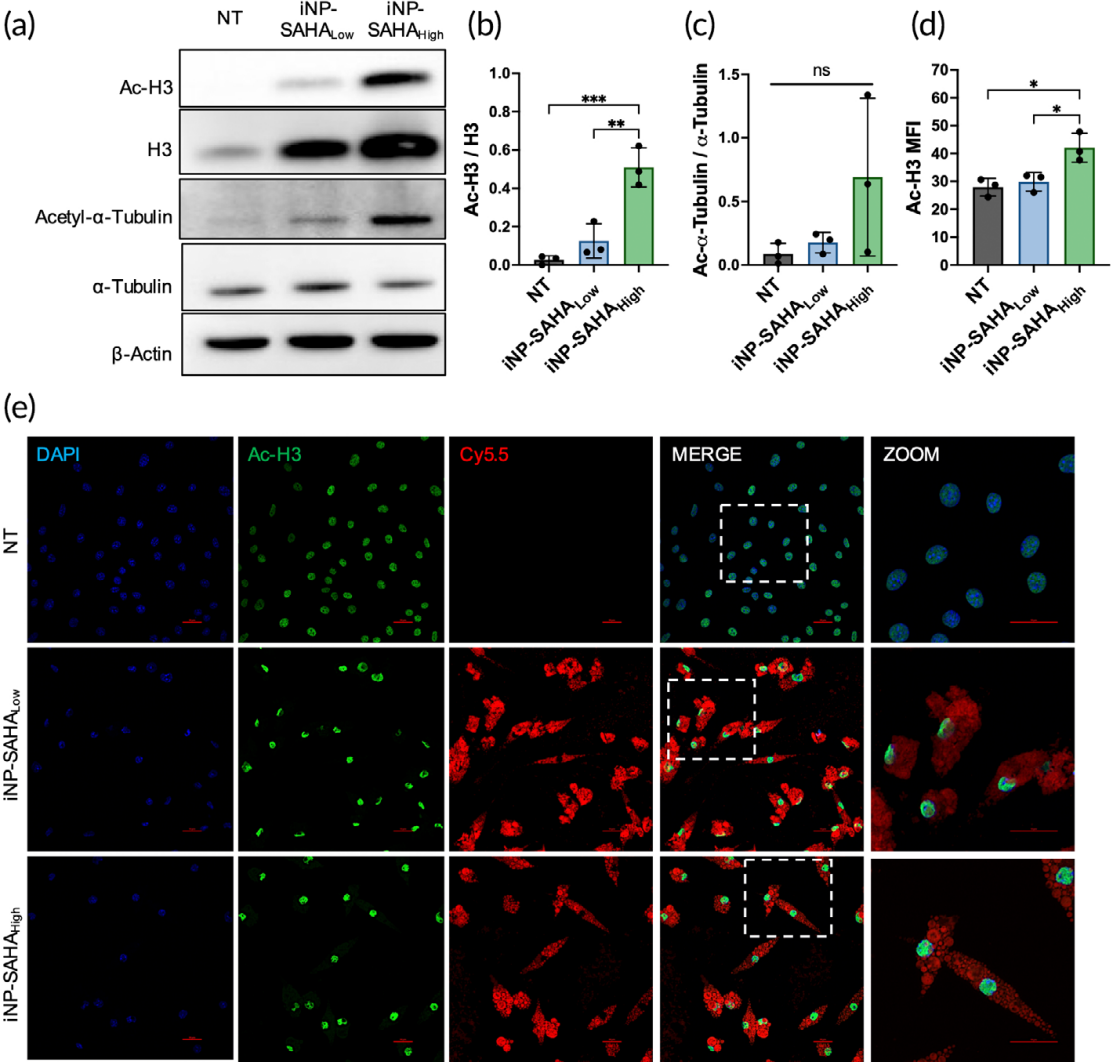
iNP‐SAHA delivered SAHA‐OH to BMMØs and acetylated histone H3 and α‐Tubulin. (a) BMMØs were pre‐treated for 3 h with iNP‐SAHA_Low_ or iNP‐SAHA_High_ at 300 μg/mL. Excess iNPs were washed with PBS and allowed to incubate for 6 h (9 h total). No treatment (NT) received no NP treatments. Cell lysates were collected, and western blot analysis used antibodies against Acetyl‐Histone H3 (Ac‐H3) and Acetylated α‐Tubulin (Ac‐α‐Tubulin). Histone H3 (H3), α‐Tubulin, and β‐Actin were used as loading controls. Quantification of the ratio of (b) Ac‐H3 to H3 and (c) Ac‐α‐Tubulin to α‐Tubulin. Immunocytochemistry (ICC) of BMMØs for DAPI (blue), Ac‐H3 (green), and cyanine 5.5 (Cy5.5, red). (d, e) BMMØs were cultured for 3 h with iNP‐SAHA_Low_‐Cy5.5, and iNP‐SAHA_High_‐Cy5.5, excess NPs were washed off, and imaged 48 h later using confocal microscopy. (d) Quantification of the mean fluorescence intensity (MFI) of the Ac‐H3 expression. (e) Representative ICC images of *n* = 3 BMMØs. Scale bars are 10 μm. One‐way ANOVA and Tukey's multiple comparisons test were performed to determine statistical differences. *p < 0.05, **p < 0.01, ***p < 0.001, and ****p < 0.0001 compared to NT control. All data are expressed as means ± SD (*n* = 3).

To confirm that the activity of SAHA‐OH was maintained when delivered from iNP‐SAHA, the induction of nuclear acetyl‐histone H3 and acetyl‐α‐tubulin in BMMØs was probed using western blotting (Figure 4a–c). BMMØs were treated for 3 h with iNP‐SAHA_Low_ and iNP‐SAHA_High_, and excess NPs were washed off and incubated for either 6 or 45 h (9 and 48 h total, respectively), then cell lysates were collected and stained for western blot analysis. iNP‐SAHA_Low_ and iNP‐SAHA_High_ treatment effectively resulted in the acetylation of histone H3 however, increased acetylation was not significantly observed for α‐tubulin despite iNP‐SAHA_High_ being increased (Figure 4b,c). Baseline acetylation levels within the no treatment (NT) group are commonly observed, as reported by other studies that have also noted similar acetylation of histone H3.^37^, ^38^ Drug‐free iNPs were used as a control and did not induce acetylation of histone H3 and α‐tubulin at similar time points (Figure S2). SAHA‐OH was similarly effective as SAHA to induce acetylation of both histone H3 or α‐tubulin at 10 μM.^17^ Next, iNP‐SAHA uptake was visualized and acetylation of histone H3 was confirmed using immunocytochemistry (ICC) (Figure 4d,e). BMMØs were treated for 3 h with iNP‐SAHA_Low_‐Cy5.5 or iNP‐SAHA_High_‐Cy5.5, then excess NPs were washed off and cells were stained and imaged 48 h later using confocal microscopy. This timepoint was chosen because our previous work showed that iNP‐Cy5.5 association with BMMØs occurs rapidly and within 3 h, where approximately 100% of the treated cells were iNP‐Cy5.5^+^ using flow cytometry.^18^ The presence of Cy5.5 within the cells confirms effective iNP internalization and z‐stacking analysis verified that iNPs were internalized rather than coating the exterior of the cells (Figure 4e, Figure S3). iNP‐SAHA treatment resulted in increased histone H3 acetylation within the nucleus as observed by an increased fluorescence signal, demonstrating delivery of active SAHA‐OH (Figure 4d). A similar experiment was also performed under LPS challenge, and the uptake of iNP‐SAHA was not significantly affected (Figure S4). Taken together, the western blot analysis in combination with ICC of BMMØs confirmed iNP‐SAHA uptake and the retention of SAHA‐OH activity following iNP‐SAHA formulation and intracellular prodrug processing.

### 3.3 iNP‐SAHA mitigates proinflammatory cytokine responses and improves cellular viability in BMMØs

**FIGURE 5:**
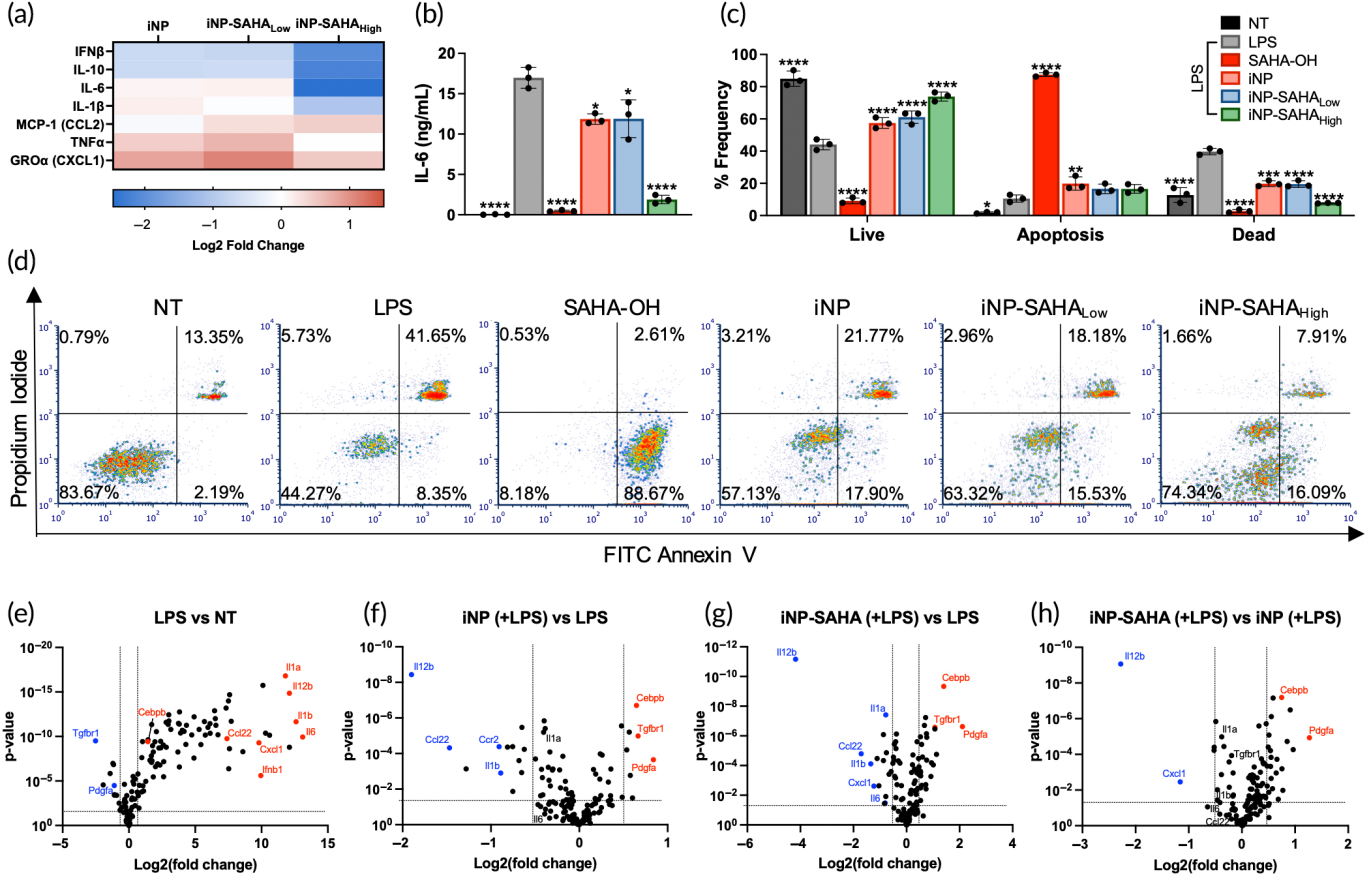
iNP‐SAHA suppressed proinflammatory cytokine secretions and apoptosis and modified gene expression profiles in vitro. BMMØs were pre‐treated with either SAHA‐OH (30 μM), iNPs (300 μg/mL), iNP‐SAHA_Low_ (300 μg/mL), or iNP‐SAHA_High_ (300 μg/mL) for 3 h. Excess iNP, iNP‐SAHA_Low_, and iNP‐SAHA_High_ were washed off with PBS. BMMØs were then subjected to LPS (100 ng/mL) stimulation for 48 h then assessed for cytokine secretions, cellular apoptosis, and gene expression. No treatment (NT) did not receive LPS stimulation or NP treatment. (a) Heat map depicting the differential cytokine secretion profiles. Two gradient‐colored heat map represents log2 fold change relative to LPS control. (b) IL‐6 cytokine measurements of BMMØs. (c) Flow cytometry analysis of cellular apoptosis was performed using FITC‐annexin V (AV) and propidium iodide (PI) where the % of live (AV^−^/PI^−^), apoptosis (AV^+^/PI^−^), and dead (AV^+^/PI^+^) BMMØs cells were plotted. (d) Representative flow cytometry plots of AV/PI staining. NanoString analysis of 248 inflammatory genes, where the volcano plots show differentially expressed genes (DEGs) comparing (e) LPS to NT, (f) iNP (+LPS) to LPS, (g) iNP‐SAHA_High_ (+LPS) to LPS, and (h) iNP‐SAHA_High_ (+LPS) to iNP (+LPS). Cut‐off at 0.5 (red) or −0.5 (blue) Log2 (fold change) and unadjusted *p*‐value <0.05. One‐way ANOVA and Tukey's multiple comparisons test were performed to determine statistical differences. *p < 0.05, **p < 0.01, ***p < 0.001, and ****p < 0.0001 compared to LPS control. All data are expressed as means ± SD (*n* = 3).

The anti‐inflammatory properties and cytotoxicity of iNP‐SAHA were examined using BMMØs following LPS challenge. BMMØs were first treated with iNP‐SAHA and iNP formulations for 3 h and excess NPs were washed away. Next, the cells were treated with 100 ng/mL LPS for 48 h before collection of the cell culture supernatants and cells for analysis using flow cytometry or NanoString gene expression analysis (Figure 5). A Luminex bead‐based immunoassay was used to investigate a set of 7 pro‐ and anti‐inflammatory cytokines and chemokines (IFNβ, IL‐10, IL‐6, IL‐1β, MCP‐1, TNFα, GROα) in the cell culture supernatants. iNP‐SAHA_High_ (equivalent to 63.75 μM SAHA‐OH) was effective at reducing IFNβ, IL‐10, IL‐6, and IL‐1β compared to iNP and iNP‐SAHA_Low_ (equivalent to 10 μM SAHA‐OH) (Figure 5a,b, Figure S5). It should be noted that the observed reduction in IL‐6 secretions by SAHA‐OH (30 μM) was attributed to the induction of apoptosis (Figure 5c,d), which was corroborated by our previous publication showing no significant apoptosis for SAHA‐OH at 10 μM but nearly 100% apoptosis for SAHA‐treated BMMØs at the same concentration.^17^ Figure 5d depicts representative flow cytometry plots of the various treatment groups assessing for live, apoptotic, and dead cells. Importantly, despite iNP‐SAHA_High_ delivering approximately twofold higher SAHA‐OH content than the soluble SAHA‐OH control, we observed a significant improvement in BMMØ viability and a significant reduction in cell death induced by LPS challenge (Figure 5c). Moreover, iNP‐SAHA_High_ also mitigated apoptosis induction that was observed in soluble SAHA‐OH (30 μM) treatment and performed similarly to iNP‐SAHA_Low_ and iNP. These findings demonstrated that iNP‐SAHA could simultaneously reduce proinflammatory responses induced by LPS and improve survival in BMMØs.

### 3.4 iNP‐SAHA modulates gene expression profiles in BMMØs

To understand how iNP‐SAHA treatment modulated the gene expression profiles of BMMØs, we employed the NanoString nCounter Mouse Inflammation v2 Panel that enabled the profiling of 248 inflammation‐related mouse genes (Figure 5e–h).^39^ The in vitro experimental design in BMMØs follows Figures 4d and 5a, as stated previously. The NanoString platform has high reproducibility, and sensitivity, and is associated with a low background signal.^40^ Volcano plots were used to identify differentially expressed genes (DEG) between various treatment groups comparing the log2 fold differences. DEGs were identified as genes with at least 0.5/−0.5 log2 fold differences and an unadjusted *p*‐value <0.05.^41^, ^42^ *p* values for NanoString data were obtained from nSolver software using a minimum count of 50 as a cutoff.^40^ A total of 134 genes met the inclusion criteria and we identified 92 DEGs for LPS versus no LPS treatment (NT), 7 DEGs for iNP (+LPS) versus LPS, 9 DEGs for iNP‐SAHA (+LPS) versus LPS, and 4 DEG for iNP‐SAHA (+LPS) versus iNP (+LPS). Compared to NT, LPS treatment significantly increased, by over 150‐fold, the expression of *Il6*, *Il‐1b*, *Il12b*, *Il‐1a*, *Ifnb1*, *Cxcl1*, *Ccl22*, and several others, while decreasing the expression of *Pdgfa* (2.16‐fold) and *Tgfbr1* (5.74‐fold) (Figure 5e). iNP treatment under LPS stimulation most significantly reduced *Il12b* (3.73‐fold) followed by *Ccl22* (2.77‐fold) compared to LPS treated BMMØs (Figure 5f). The reduction of *Il12b* was significantly potentiated using iNP‐SAHA treatment under LPS stimulation (18.13‐fold) compared to LPS treated BMMØs (Figure 5g). *Ccl22* (3.27‐fold) and *Il1b* (2.55‐fold) were also further reduced while *Pdgfa* (4.32‐fold) and *Cebpb* (2.64‐fold) was increased in iNP‐SAHA treatment versus LPS, but only modestly greater than iNP groups. Additionally, iNP‐SAHA and iNP under LPS challenge had slight effects (<2.1‐fold) on *Tgfbr1*, *Il‐1a*, and *Il‐6*. Finally, comparing iNP‐SAHA to iNP treated BMMØs under LPS stimulation, we identified significant reductions in *Il12b* (4.86‐fold) and *Cxcl1* (2.23‐fold) expression, while *Pdgfa* (2.41‐fold) was increased (Figure 5h). Overall, the most remarkable reductions in gene expression were observed for *Il12b* and *Cxcl1*, indicating the potential of iNP‐SAHA in suppressing proinflammatory responses. Conversely, *Pdgfa* showed overall increases, indicating that iNP‐SAHA may be useful to promote wound healing and tissue repair processes. These findings shed light on the intricate molecular interactions that contribute to iNP‐SAHA's therapeutic effects in mitigating severe inflammation and sepsis‐induced immune dysfunction.

### 3.5 iNP are retained locally following intraperitoneal injection

**FIGURE 6:**
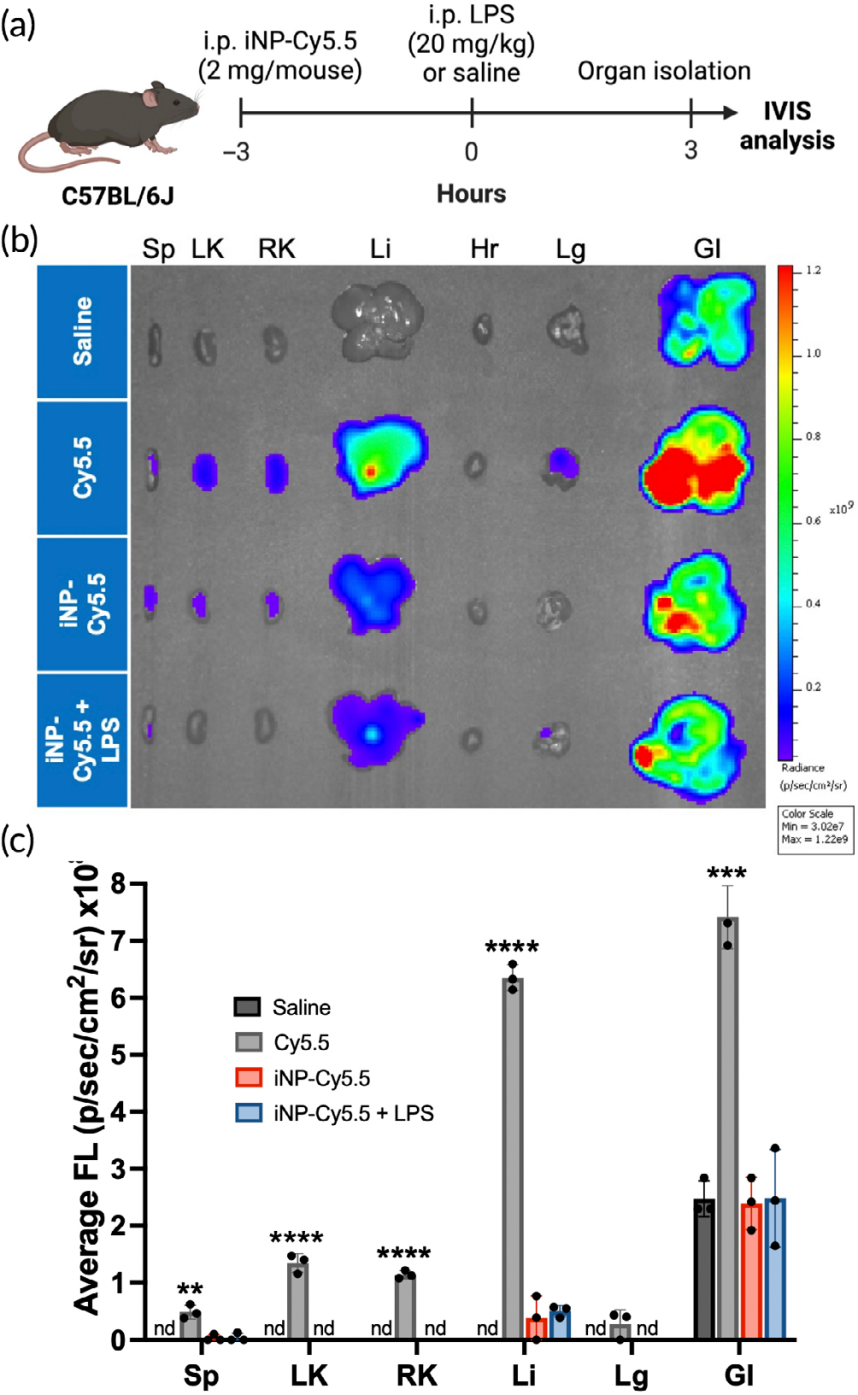
Biodistribution of iNP‐Cy5.5 in a prophylactic LPS‐induced endotoxemia mouse model. (a) In vivo i.p. dosing regimen where mice were pre‐treated with either saline, soluble PLGA‐Cy5.5 conjugates (Cy5.5), or iNP containing Cy5.5 (iNP‐Cy5.5) (2 mg/mouse) for 3 h. Subsequently, mice were then subjected to either saline or 20 mg/kg LPS challenge for 3 h. (b) Spleen (Sp), left and right kidneys (LK, RK), liver (Li), heart (Hr), lung (Lg), and GI tract (GI) (from left to right) were isolated and analyzed via in vivo imaging systems (IVIS). (c) Average fluorescence intensity (FL) was quantified from the region of interest (ROI). No fluorescence intensity of the hearts was detected. One‐way ANOVA and Tukey's multiple comparisons test were performed to determine statistical differences. *p < 0.05, **p < 0.01, ***p < 0.001, and ****p < 0.0001 compared to Saline control. All data are expressed as means ± SD (*n* = 3). nd, not detected.

We examined the biodistribution of iNPs in vivo by formulating iNPs to incorporate cyanine 5.5 (Cy5.5), a fluorescent dye (Table S1). Mice were administered i.p. injections of soluble PLGA‐Cy5.5 (herein termed Cy5.5) or iNP containing PLGA‐Cy5.5 (iNP‐Cy5.5), then 3 h later, subjected to either i.p. saline (control) or LPS challenge (20 mg/kg) (Figure 6a). Various organs (spleen, left and right kidney, liver, heart, lungs, and GI tract) were isolated 3 h later and imaged using IVIS (Figure 6b,c). It was observed that Cy5.5 distributed to a larger extent than iNP‐Cy5.5, as observed with higher fluorescent intensities in the various organs. The lower Cy5.5 fluorescence in the liver, spleen, and kidneys via iNP delivery is not unexpected, as the size of iNP‐Cy5.5 was approximately 600 nm and similar NPs have been shown to interact with immune cells, which may result in reduced systemic exposure.^43^, ^44^ Overall, the data showed that iNPs are locally retained and the presence of systemic inflammation does not significantly alter their biodistribution.

### 3.6 iNP‐SAHA and iNP provide a dose‐dependent survival protection against LPS‐induced endotoxemia

**FIGURE 7:**
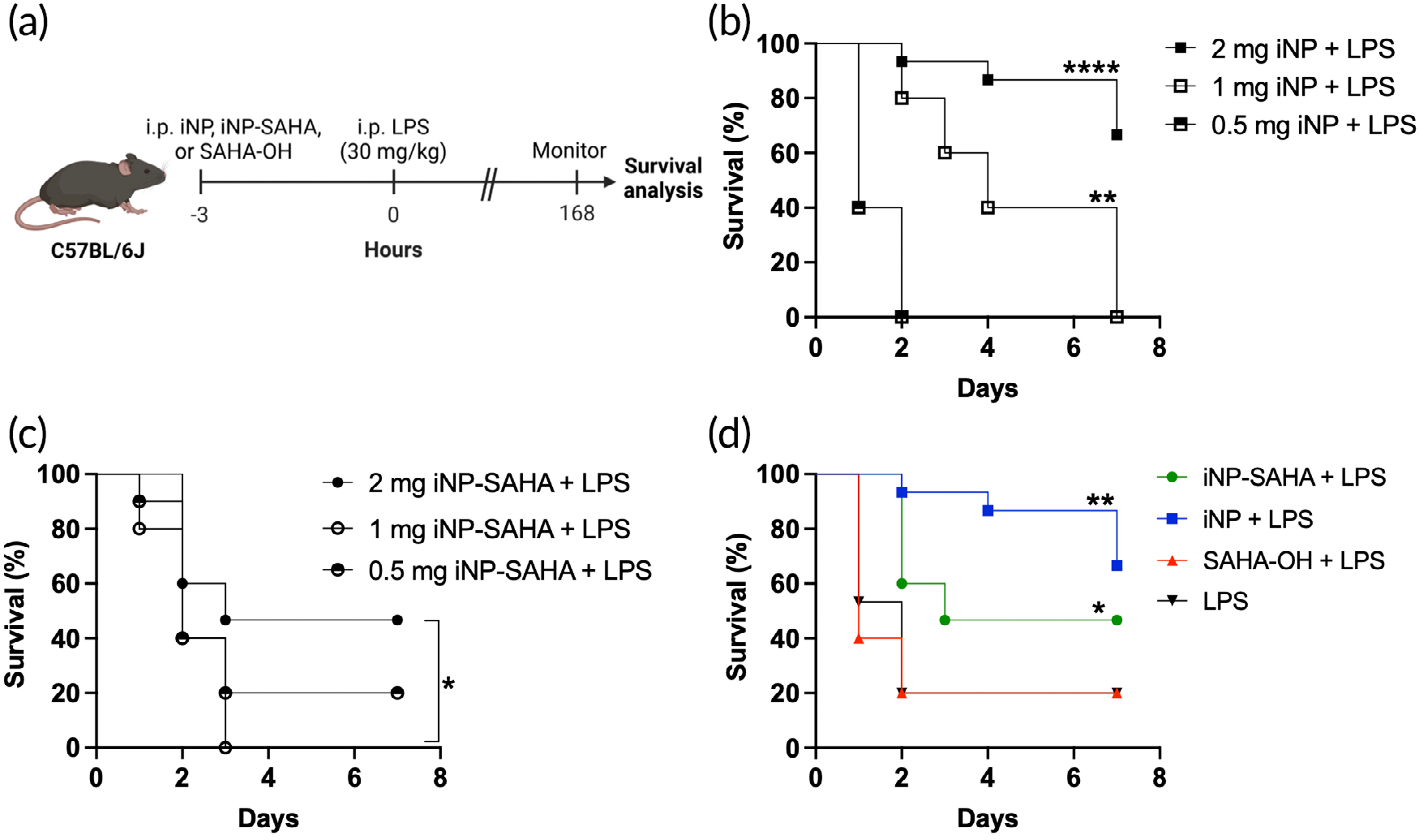
Dose‐dependent survival of iNP and iNP‐SAHA in a prophylactic LPS‐induced endotoxemia mouse model. (a) In vivo i.p. dosing regimen of iNP, iNP‐SAHA, or 50 mg/kg SAHA‐OH treated 3 h prior to 30 mg/kg LPS challenge in a prophylactic LPS‐induced endotoxemia C57BL/6 male mice model. Mice were monitored for 7 days (168 h) to assess survival. (b) Dose escalation studies of 2 mg iNP (*n* = 15), 1 mg iNP (*n* = 5), and 0.5 mg iNP (*n* = 5) prior to LPS challenge. (c) Dose escalation studies of 2 mg iNP‐SAHA_Low_ (*n* = 15), 1 mg iNP‐SAHA_Low_ (*n* = 10), or 0.5 mg iNP‐SAHA_Low_ (*n* = 10) prior to LPS challenge. (d) Mice were administered 2 mg/mouse iNP‐SAHA_Low_ (*n* = 15) or 2 mg/mouse iNP (*n* = 15) or 50 mg/kg SAHA‐OH (*n* = 5) prior to LPS treatment. Control LPS mice (*n* = 15) subjected to only 30 mg/kg LPS treatment and pre‐treated with saline (20 μL/g body weight). Kaplan–Meier curves and log‐rank (Mantel‐Cox) tests were performed to compare the survival rates. *p < 0.05, **p < 0.01, ***p < 0.001, and ****p < 0.0001.

Based on the low toxicity and overall reductions in proinflammatory cytokine secretions induced by iNP‐SAHA_High_ (termed iNP‐SAHA) (Figure 5), this NP formulation was chosen for further evaluation of its therapeutic efficacy in vivo. The survival benefits of iNP‐SAHA treatment were assessed using a lethal LPS‐induced endotoxemia mouse model.^18^ The lethal dose of LPS was determined as 30 mg/kg for use in the current studies (Figure S6). We investigated the dose‐dependent survival effects by evaluating three doses of iNP and iNP‐SAHA (0.5, 1, and 2 mg/mouse). Briefly, mice were administered iNP‐SAHA (*n* = 15) or iNP (*n* = 15) via i.p. injection 3 h before 30 mg/kg LPS and the survival was tracked over 7 days (Figure 7a). As a control, mice subjected to LPS only were pretreated with saline (*n* = 15). For both NP types, the 2 mg dose was most effective at protecting against LPS‐induced mortality, with lower doses being less effective (Figure 7b,c). Forty‐seven percent of iNP‐SAHA‐treated mice (2 mg) survived following LPS challenge, while 67% of iNP‐treated mice (2 mg) survived, compared to the control SAHA‐OH‐treated mice (20% survival) or the control LPS‐treated mice (20% survival) (Figure 7d). Overall, iNP and iNP‐SAHA improved survival outcomes in a dose‐dependent manner as compared to SAHA‐OH treatment but did not perform significantly different from each other in the LPS‐induced endotoxemia model, despite previous studies from Li et. al showing treatment with SAHA can improve survival, before and after LPS challenge.^38^, ^45^ These results prompted further investigation of the differences in plasma cytokines and organ histology to better understand the differences between iNP‐SAHA and iNP effects.

### 3.7 iNP‐SAHA reduced plasma proinflammatory cytokine levels and demonstrated organ biocompatibility

**FIGURE 8:**
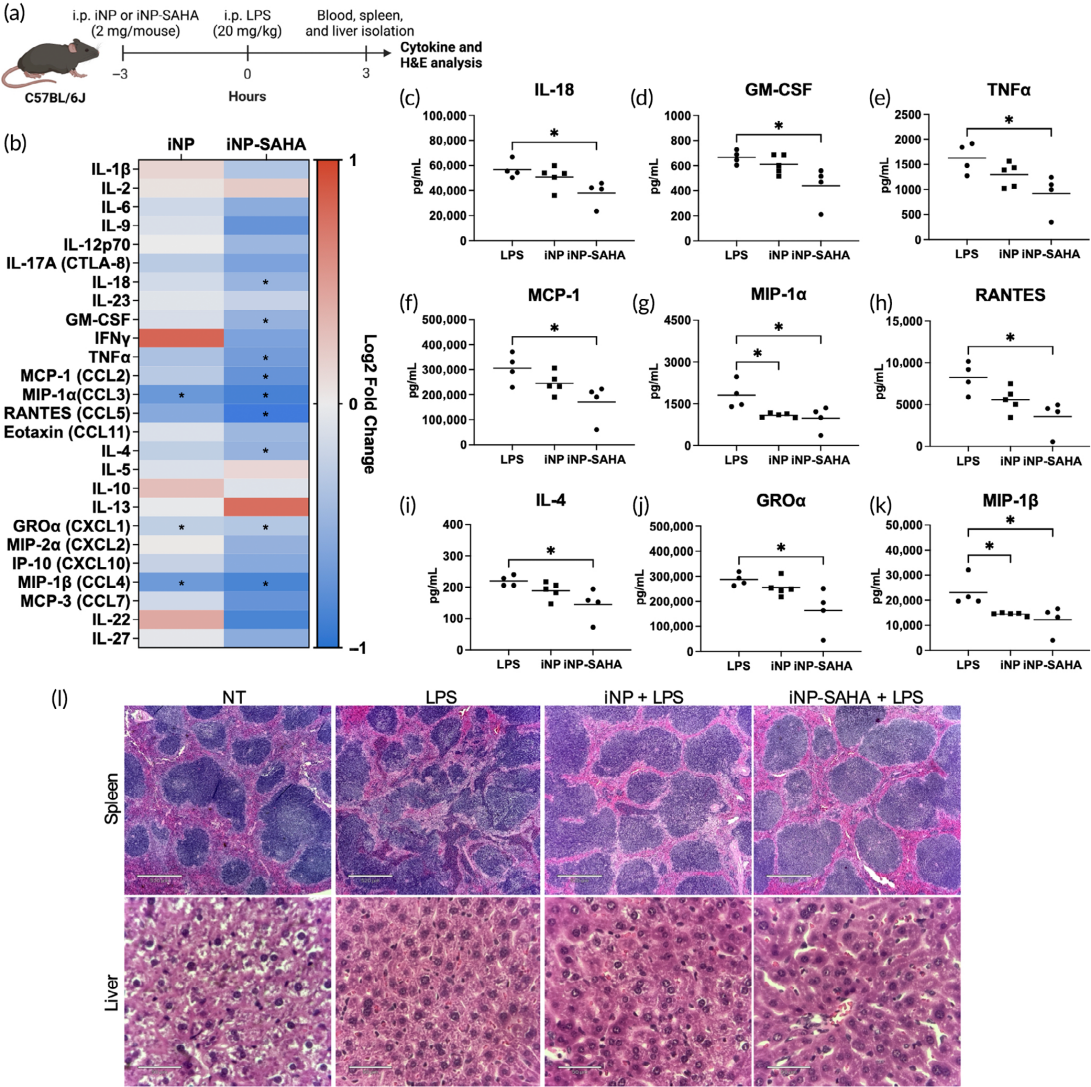
iNP‐SAHA reduced plasma levels of proinflammatory cytokines and is biocompatible in mouse organ systems. (a) In vivo i.p. dosing regimen of iNP (2 mg/mouse) or iNP‐SAHA (2 mg/mouse) treated 3 h prior to 20 mg/kg LPS challenge in a prophylactic LPS‐induced endotoxemia C57BL/6 male mice model. LPS control was treated with saline (20 μL/g body weight) for 3 h and subjected to 20 mg/kg LPS i.p. injections. No treatment (NT) received no LPS challenge or NP treatment. The blood, spleen, and liver were collected 3 h later for assessment. (b) Multiplex Luminex analysis of 26 cytokines and chemokines was performed on mouse plasma. Log2 fold change relative to LPS is represented in a two gradient color heat map. Differential suppression of (c) IL‐18, (d) GM‐CSF, (e) TNFα, (f) MCP‐1, (g) MIP‐1α, (h) RANTES, (i) IL‐4, (j) GROα, and (k) MIP‐1β. (l) H&E stains of spleen and liver slices. Scale bar for spleen slices is 520 μm. Scale bar for liver slices is 50 μm. One‐way ANOVA and Tukey's multiple comparisons test were performed to determine statistical differences. *p < 0.05. All data are expressed as mean ± SD (*n* = 4 or 5).

To examine the systemic effects of iNP‐SAHA and iNP treatment, mice were administered 2 mg i.p./mouse, 3 h before 20 mg/kg LPS. LPS control mice were injected with saline 3 h before LPS challenge. Three hours post‐LPS, the mice were euthanized, and the plasma, spleen, and liver were isolated (Figure 8a). Multiplex analysis by Luminex assessing for 26 various cytokines and chemokines was performed to measure the systemic immunomodulatory effects of iNP‐SAHA (Figure 8b).^46^ Local (i.p.) administration of iNP‐SAHA significantly reduced plasma levels of multiple cytokines and chemokines, including IL‐18, GM‐CSF, TNFα, MCP‐1, MIP‐1α, RANTES, IL‐4, GROα, and MIP‐1β, compared to LPS‐treated mice (Figure 8c–k). iNP‐SAHA was more effective at modulating cytokine responses than iNPs, where iNPs only reduced 2 chemokines, MIP‐1α and MIP‐1β, compared to LPS‐treated mice. These mediators are characteristic markers of sepsis, and their elevated levels can contribute to sepsis progression through the activation of innate immune responses and recruitment of inflammatory immune cells.^2^ The suppression of these proinflammatory markers indicated that iNP‐SAHA could mitigate the acute proinflammatory phase of sepsis through a multimodal anti‐inflammatory mechanism.

To establish the impact of iNP‐SAHA treatment on organ architecture, histological examination via H&E staining was performed (Figure 8l). Control spleen and liver sections from the no treatment (NT) mice had well‐defined organ architecture and overall unaltered histopathology. Mice subjected to LPS challenge presented ill‐defined spleen sections with distorted white pulp margins and dysregulated germinal centers.^47^ The spleen and liver histological samples from iNP‐SAHA and iNP‐treated mice showed similar histopathology compared to the control NT mice, demonstrating biocompatibility and potential inhibition of inflammation‐induced organ damage. At the current time point, no observed histological differences were observed in any liver sections among the various treatment groups. These findings support the safety and potentially multimodal immunomodulatory effects of iNP‐SAHA and iNP administration.

## 4 DISCUSSION

Sepsis is a complex and multifaceted disease for which there is no cure. Current treatment strategies mainly focus on infection control and supportive care such as the administration of fluids and vasopressors. Given the lack of an FDA‐approved treatment option that improves patient survival and the limited success of clinical trials targeting single molecular pathways, we developed iNP‐SAHA as a potential polypharmacological strategy to target the acute proinflammatory response, improve cell survival, and ultimately reduce sepsis symptoms and improve survival.^4^, ^48^, ^49^, ^50^, ^51^ The iNP‐SAHA platform is built upon two recent findings from our group: (1) inherently anti‐inflammatory and drug‐free iNPs,^18^, ^20^, ^21^ and (2) SAHA‐OH, a novel HDACi analog that displays potent anti‐inflammatory properties and an improved toxicity profile compared to the pan‐HDACi SAHA (Figure 1).^17^ SAHA‐OH was precisely incorporated into iNPs at two distinct loadings by first creating a PLGA‐SAHA polymer conjugate (ester prodrug) followed by combining with unmodified PLA prior to iNP‐SAHA formation using a single emulsion‐solvent evaporation method (Figure 3a, Figure S1). This approach enabled stoichiometric incorporation of SAHA‐OH into iNP‐SAHA and tailored drug delivery and distribution to organs and innate immune cells targeted by iNPs (Figures 1, 3, and 6, Figure S1).^18^, ^52^ Using a polymer‐drug conjugation approach allows for tunable drug loading, enables controlled drug release, and reduces SAHA‐OH drug‐associated side effects.^53^ Two formulations of iNP‐SAHA were prepared, iNP‐SAHA_Low_ (containing 10 μM of SAHA‐OH) and iNP‐SAHA_High_ (containing 63 μM of SAHA‐OH), where 10 μM was selected based on our previous publication where SAHA‐OH showed distinct differences in toxicity compared to other concentrations.^17^ To further explore the potential for enhanced anti‐inflammatory effects, we investigated the loading‐dependent impact of SAHA‐OH within iNP‐SAHA. We prepared iNP‐SAHA with the maximum possible loading of SAHA‐OH (63 μM), which was determined based on the weight ratio of SAHA‐OH to PLGA in the PLGA‐SAHA conjugate. Moreover, the utilization of drug delivery systems to incorporate SAHA has proven crucial in overcoming inherent limitations of the drug, such as poor water solubility, short half‐life, and toxicity.^54^, ^55^

Delivery of SAHA‐OH from iNP‐SAHA was confirmed by measuring histone H3 acetylation within the nucleus and α‐tubulin acetylation within the cytoplasm (Figure 4), which was not observed in the iNP control and was iNP‐SAHA loading‐dependent (Figure S2). It is possible that SAHA‐OH was not fully released from the PLGA‐SAHA conjugate by degradation; however, the conjugation of SAHA‐OH to PLGA occurs at capping group of SAHA‐OH that is not required for HDAC binding (Figure 2).^56^, ^57^ Since our studies showed nuclear histone H3 acetylation, it is more likely that SAHA‐OH was liberated because confocal image analysis did not show the presence of iNP‐SAHA in the nucleus (Figure 4e). Taken together, the incorporation of SAHA‐OH into iNPs allowed for precise delivery to target cells, leading to the successful processing, and retained biological activity of SAHA‐OH.

Previous studies from our lab have shown that iNPs employ a multimodal mechanism of action to mitigate the induction of proinflammatory responses that consists of a physical blockade of LPS interactions with cell membranes and functional reprogramming of NF‐κB p65 and p38 MAPK signaling.^20^ Here, the modulation of NF‐κB and p38 MAPK signaling by iNPs was suggested by measuring significant reductions in proinflammatory cytokine secretions from macrophages under LPS stimulation, such as IFNβ and IL‐6 (Figure 5a,b, Figure S5F). Of note, *Nfkb1* expression, which encodes for NF‐κB p105 and p50, showed minimal expression differences following iNP or iNP‐SAHA treatment (Figure 5f).^58^ The immunomodulatory effects of iNP most likely stems from its ability to modify the phosphorylation of NF‐κB p65, rather than downregulating *Nfkb1* encoding for NF‐κB p105/p50.^20^ Corroborating the current study, intravenous administration of iNP followed by isolation of splenocytes and subsequent stimulation with LPS or CpG ODN resulted in significant decreases in IL‐6, MCP‐1, and TNFα secretions.^18^ The addition of SAHA‐OH into iNPs may have further contributed to the downregulation in NF‐κB and p38 MAPK, offering synergistic anti‐inflammatory effects.^14^ This was observed in Figure 5a,b, where iNP‐SAHA_High_ treatment under LPS stimulation resulted in significant reductions in various proinflammatory and anti‐inflammatory mediators regulated by NF‐κB, such as IFNβ, IL‐6, IL‐1β, and IL‐10.^59^ Indeed, the ability of iNP‐SAHA to mitigate proinflammatory cytokine secretions at this early time point is beneficial in overcoming the initial cytokine storm.^60^ IL‐10, an anti‐inflammatory cytokine, can play a contextual role where lower levels initially can enhance survival through the promotion of infection control, while prolonged IL‐10 deficiencies can result in susceptibility to nosocomial infections and immunosuppression.^61^ SAHA‐OH at a concentration of 10 μM was deemed safe and effective due to the alleviation of SAHA‐induced apoptosis in primary macrophages and maintained anti‐inflammatory function.^17^ This same concentration of SAHA‐OH used in iNP‐SAHA_Low_ (equivalent to 10 μM SAHA‐OH) did not significantly reduce proinflammatory cytokine levels as compared to iNP control (Figure 5a,b). This led to the generation of a formulation with a higher loading of SAHA‐OH, iNP‐SAHA_High_, equivalent to 63.75 μM SAHA‐OH (Figure 3a). Remarkably, the higher loaded formulation of iNP‐SAHA_High_ beneficially improved macrophage viability and reduced SAHA‐OH‐induced apoptosis at higher concentrations. This contrasted with our previous publication, where SAHA‐OH at 30 μM induced apoptosis in primary macrophages (Figure 5c,d). This demonstrated the effective use of NP platforms to improve drug loading and reduce drug‐associated toxicities while maintaining beneficial anti‐inflammatory functions.

The assessment of gene expression was performed using the NanoString nCounter system that allowed for the direct measurement of mRNA expression levels in a highly sensitive, precise, and reproducible manner.^39^, ^40^ The treatment of iNP‐SAHA_High_ (referred to as iNP‐SAHA), as compared to either iNP or LPS treatment, resulted in the modulation of several genes, including *Il‐12b*, *Cxcl1*, *Pdgfa*, and *Ccl22* (Figure 5f–h). IL‐12b, also known as IL‐12p40, is a proinflammatory cytokine that regulates immune defense by inducing cytotoxic lymphocytes and enhancing natural killer cell cytotoxicity.^62^ Notably, iNP‐SAHA significantly reduced *Il‐12b* expression by 18.3‐fold (Figure 5g,h), which was upregulated in LPS‐stimulated primary macrophages (Figure 5e), and has been observed in a polymicrobial murine model of abdominal sepsis and human sepsis.^62^, ^63^ Additionally, iNP‐SAHA treatment significantly reduced *Cxcl1* expression, where CXCL1 is an inflammatory chemokine that regulates neutrophil recruitment.^64^ Increased expression of *Pdgfa* upon iNP‐SAHA treatment could have implications in improving wound healing and tissue repair, as platelet‐derived growth factor A (PDGFA) has shown to play a role in monocyte–macrophage proliferation and chemotaxis.^65^ Furthermore, lowered expression of *Ccl22* upon iNP‐SAHA treatment could result in reduced immune cell infiltration during inflammation.^66^ This demonstrates the multimodal ability of iNP‐SAHA to modulate various gene pathways that are dysregulated under inflammatory conditions.

The prior in vitro studies showed that iNP‐SAHA has beneficial anti‐inflammatory properties and improves cellular survival. iNP‐SAHA was evaluated using an in vivo mouse model of sepsis using LPS‐induced endotoxemia.^18^ Loading of SAHA‐OH into polymer‐based nanoparticles has been shown to be biocompatible in mouse models and to improve drug delivery and distribution to various organs.^55^ Nanoparticles are rapidly cleared to the liver and spleen via the mononuclear phagocyte system and reticuloendothelial system following intravenous administration.^67^ In this study, iNP‐SAHA‐Cy5.5 was locally i.p. administered and distributed to the gastrointestinal (GI) tract, liver, spleen, and kidneys (Figure 6b,c).^43^ Furthermore, treatment with iNP‐SAHA showed dose‐dependent improvements in survival in a lethal LPS‐endotoxemia mouse model of sepsis (Figure 7c,d, Figure S6). Previous studies have shown improved survival efficacy of i.p. administered iNPs in the same prophylactic endotoxemia mice model.^18^ Interestingly, iNP‐SAHA did not show significant differences in survival compared to iNP‐treatment, which was contrary to the in vitro observations. This prompted further analysis of the systemic cytokine profile and organ histology. Locally administered iNP‐SAHA was highly effective at reducing the systemic proinflammatory cytokine plasma levels compared to iNP treatment (Figure 8b–k). Inhibition of plasma TNFα was observed following i.p. iNP‐SAHA administration (Figure 8e), but IL‐6 was not significantly reduced in vivo, contrary to the in vitro assays (Figure 5b). Previous studies using human monocytes have found that high concentrations of lactic acid (LA) impacted TNFα levels more than IL‐6, suggesting that LA may reduce TNFα but not modulate IL‐6 secretion under in vivo conditions.^23^ Finally, histological analysis showed improved histopathological architecture in spleen or liver organ structure in mice treated with iNP or iNP‐SAHA under LPS challenge, compared to LPS control mice (Figure 8l). Overall, the mechanisms underlying sepsis survival are highly intricate, involving multiple interconnected biological pathways that extend beyond the cytokine profile, and the clinical heterogeneity of the disease adds unique complexities to the development of immunotherapies.^4^, ^5^, ^51^, ^68^ While iNP‐SAHA did not exhibit significant survival improvements compared to iNP, it demonstrated notable enhancements in survival when compared to LPS control mice. Moreover, iNP‐SAHA showed remarkable ability to reduce systemic cytokine levels when locally administered, surpassing the performance of iNP treatment. The anti‐inflammatory properties of iNP‐SAHA are promising, warranting further investigation in other models of severe inflammation, such as rheumatoid arthritis (RA) or inflammatory bowel disease (IBD), which represent localized inflammation scenarios in contrast to the systemic inflammation seen in sepsis. These findings affirm the safety, tolerability, and effectiveness of iNP‐SAHA in mitigating acute hyperinflammation and offering protection against LPS‐induced septic shock in animal models. This encourages exploration of iNP‐SAHA's therapeutic potential in diverse inflammatory disorders beyond sepsis.

The present study focused on the development and investigation of the potential multimodal biological effects of iNP‐SAHA. Incorporation of SAHA‐OH into iNP resulted in an improved toxicity profile, while significantly reducing LPS‐induced proinflammatory responses in primary macrophages. Our in vivo studies were performed using a prophylactic mouse model of LPS‐induced endotoxemia and the limitation of evaluating the efficacy of iNP‐SAHA using this treatment regimen should be acknowledged. This approach was motivated by the findings of Casey et al., who reported that iNPs were more effective to improve mouse survival when administered prophylactically rather than therapeutically.^18^ Considering that iNPs have been previously found to not sequester LPS,^20^ it is unlikely that iNP‐SAHA would be capable of modulating LPS‐induced mortality using a therapeutic model. It is also important to note that the LPS‐induced endotoxemia mouse model, which is widely used in sepsis research as a model of the cytokine storm, has inherent limitations in fully replicating the complex and diverse dysregulated responses seen in human sepsis.^69^ On the other hand, the cecal ligation and puncture (CLP) mouse model of polymicrobial peritonitis is considered to be a clinically relevant model of sepsis as it mimics the polymicrobial nature of human sepsis and involves a more dynamic and complex interplay between the host and the invading pathogens.^70^ Therefore, it is important to interpret the findings of the current study in the context of the specific model used. Future experimental work using the CLP model may provide valuable insights into the potential therapeutic efficacy of iNP‐SAHA in a more clinically relevant setting for sepsis treatment.

## 5 CONCLUSION

In conclusion, the presented work demonstrates the anti‐inflammatory benefits of iNP‐SAHA to attenuate acute hyperinflammation, improve cell survival, and protect against LPS‐induced inflammation using in vitro and in vivo models. The multimodal immunomodulatory effects of iNP‐SAHA could aid in mitigating the cytokine storm associated with sepsis and reducing systemic inflammation. These results suggest that iNP‐SAHA could have potential translational implications for sepsis in humans, a condition with high mortality and limited treatment options. Although the scope of this study primarily focuses on acute hyperinflammation, future work determining the impact of iNP‐SAHA on long‐term effects of sepsis is warranted, such as wound repair and apoptosis‐induced immunosuppression.

## AUTHOR CONTRIBUTIONS

**Nhu Truong:** Conceptualization (equal); formal analysis (lead); funding acquisition (supporting); investigation (lead); visualization (lead); writing – original draft (equal); writing – review and editing (lead). **Andrea L. Cottingham:** Investigation (supporting). **Shruti Dharmaraj:** Investigation (supporting). **Jacob R. Shaw:** Investigation (supporting). **Jackline Joy Martin Lasola:** Investigation (supporting). **Christopher C. Goodis:** Resources (supporting). **Steven Fletcher:** Resources (lead). **Ryan M. Pearson:** Conceptualization (equal); formal analysis (supporting); funding acquisition (lead); supervision (lead); writing – original draft (equal); writing – review and editing (supporting).

## CONFLICT OF INTEREST STATEMENT

The authors declare the following competing financial interest(s): Ryan M. Pearson, Steven Fletcher, and Nhu Truong are inventors on a patent application that describes SAHA‐OH and iNP‐SAHA technology.

### PEER REVIEW

The peer review history for this article is available at https://www.webofscience.com/api/gateway/wos/peer-review/10.1002/btm2.10611.

## Supporting information


**Data S1.** Supporting Information.

## Data Availability

Data are available upon request from the authors.
