# Supplementary material for: Multimodal nanoparticle‐containing modified suberoylanilide hydroxamic acid polymer conjugates to mitigate immune dysfunction in severe inflammation
Source: Bioeng Transl Med. 2023 Oct 14;9(1):e10611. doi: 10.1002/btm2.10611 (PMC10771562; doi:10.1002/btm2.10611)
Supplement: Supplementary file 1 — Data S1. Supporting Information. [file BTM2-9-e10611-s001.docx]

*Supporting Information*

**Multimodal nanoparticle-containing modified suberoylanilide hydroxamic acid polymer conjugates to mitigate immune dysfunction in severe inflammation**

Nhu Truong,^1^ Andrea L. Cottingham,^1^ Shruti Dharmaraj,^1^ Jacob R. Shaw,^2^ Jackline Joy Martin Lasola,^2^ Christopher C. Goodis,^1^ Steven Fletcher,^1^ Ryan M. Pearson^1,2,3,^**^*^**

1. Department of Pharmaceutical Sciences, University of Maryland School of Pharmacy, 20 N. Pine Street, Baltimore, MD 21201.
2. Department of Microbiology and Immunology, University of Maryland School of Medicine, 685 W. Baltimore Street, Baltimore, MD 21201.
3. Marlene and Stewart Greenebaum Comprehensive Cancer Center, University of Maryland School of Medicine, 22 S. Greene Street, Baltimore, MD 21201.

**^*^Address correspondence to:**

Ryan M. Pearson, Ph.D.

Department of Pharmaceutical Sciences

University of Maryland School of Pharmacy

20 N. Pine Street

N525 Pharmacy Hall

Baltimore, MD 21201

Phone: 410-706-3257

Email: [rpearson@rx.umaryland.edu](mailto:rpearson@rx.umaryland.edu)

**Figure S1.** (A) Schematic of the nanoformulation of PLGA-SAHA prodrug and unmodified PLA at specific (B) stoichiometric ratios to synthesize two loadings of iNP-SAHA via o/w single emulsion technique.

**A**

**B**


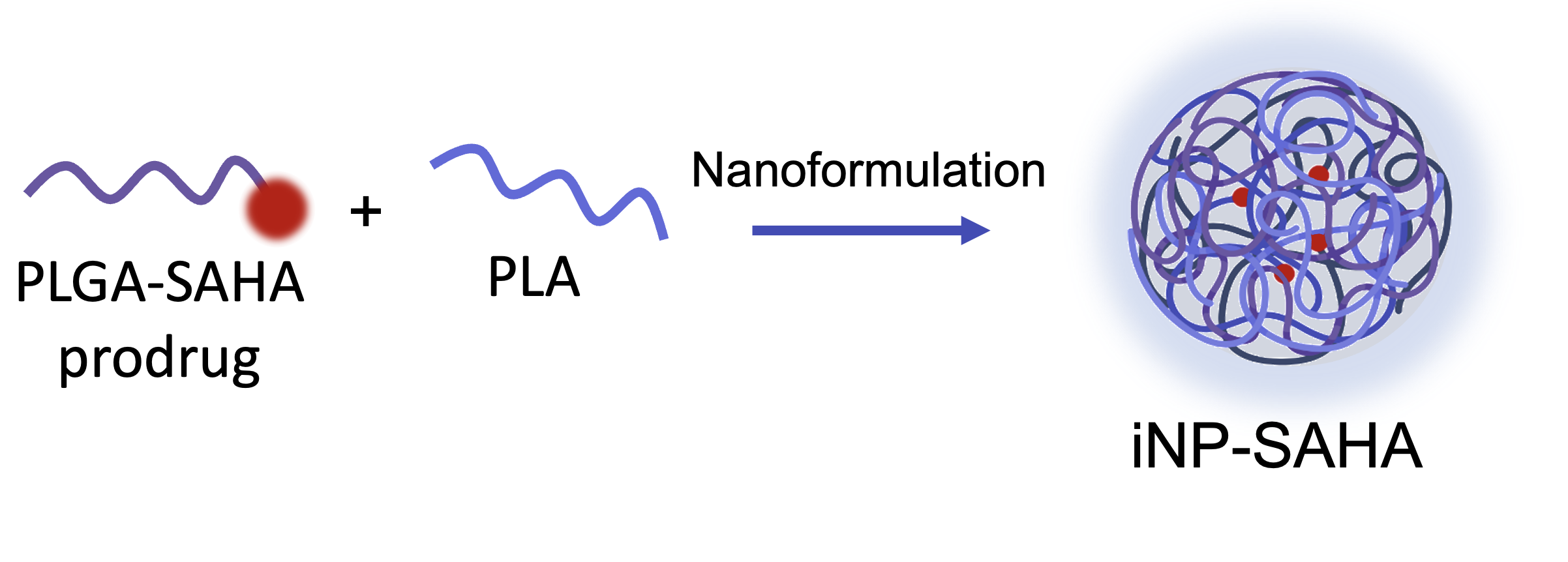

**Table S1.** iNP formulations with Cy5.5 were synthesized for imaging studies containing none, low, and high SAHA loading. DLS characterization determined the particle size (nm), polydispersity index (PDI), and zeta potential (mV).

**Figure S3. iNP do not acetylate histone H3 or ⍺-Tubulin.** BMMØs were pre-treated for 3 hours with cargo-less iNPs at 300 µg/mL. Excess iNPs were washed with PBS and allowed to incubate for either 1, 6, or 24 hours (4 hours, 9 hours, and 27 hours, respectively). No treatment (NT) received no NP treatment. Cell lysates were collected, and western blot analysis used antibodies against acetylated histone H3 and acetylated ⍺-tubulin. Histone H3 and ⍺-Tubulin was used as loading controls.

**Figure S2. iNP do not acetylate histone H3 or ⍺-Tubulin.** BMMØs were pre-treated for 3 hours with cargo-less iNPs at 300 µg/mL. Excess iNPs were washed with PBS and allowed to incubate for either 1, 6, or 24 hours (4 hours, 9 hours, and 27 hours, respectively). No treatment (NT) received no NP treatment. Cell lysates were collected, and western blot analysis used antibodies against acetylated histone H3 and acetylated ⍺-tubulin. Histone H3 and ⍺-Tubulin was used as loading controls.


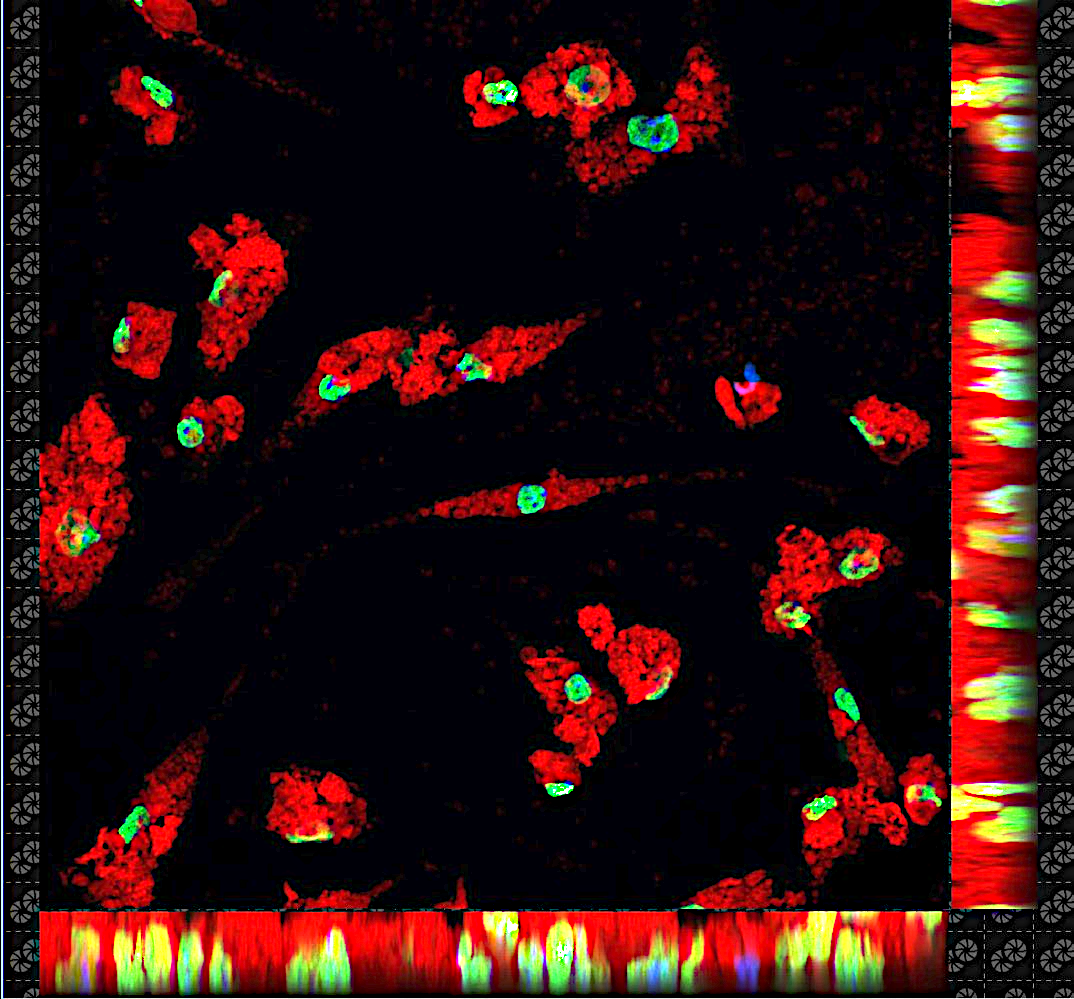


**Figure S3. iNP-SAHA is internalized in BMM∅s.** Immunocytochemistry (ICC) of BMM∅s stained for DAPI and acetylated histone H3. BMM∅s were cultured for 3 hours with iNP-SAHA_Low_-Cy5.5, excess NPs were washed off, and imaged 48 hours later via confocal microscopy. Z-stacked images demonstrate internalization of iNP-SAHA_Low_ within the cell.

**Figure S4. Uptake of iNP-SAHA in BMM∅s under inflammatory conditions.** Immunocytochemistry (ICC) of BMMØs for DAPI (blue), acetylated histone H3 (Ac-Histone H3, green), and cyanine 5.5 (Cy5.5, red). BMM∅s were cultured for 3 hours with iNP-SAHA_Low_-Cy5.5, excess NPs were washed off, and imaged 48 hours later via confocal microscopy. To simulate an inflammatory state, BMM∅s were cultured for 3 hours with iNP-SAHA_Low_-Cy5.5, excess NPs were washed off, subjected to 300 ng/mL LPS stimulation, and imaged 48 hours later via confocal microscopy. No treatment (NT) received no LPS stimulation or NP treatment. Images are representative of n=3 BMMØs. Scale bars are 10 µm.


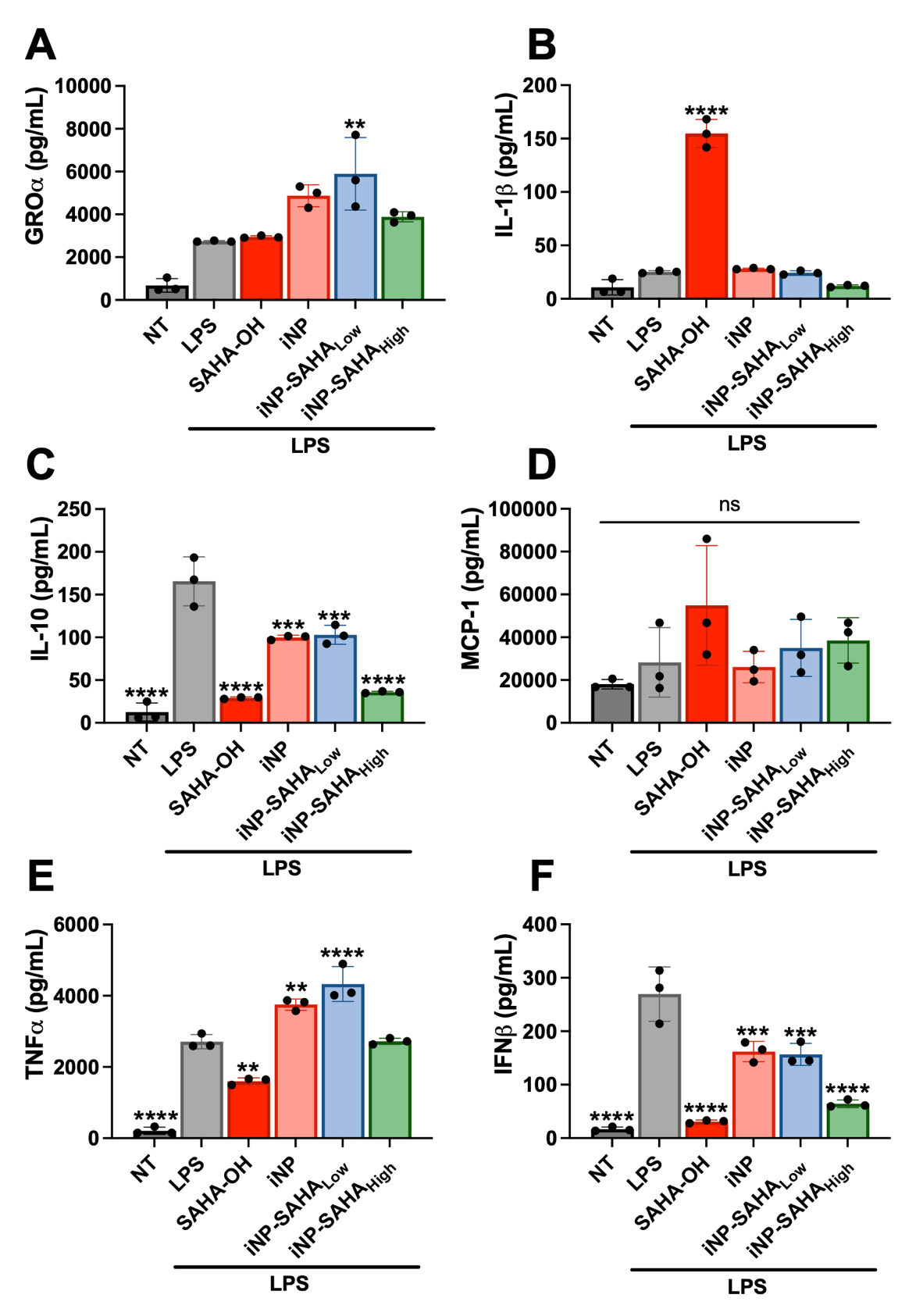


**Figure S5.** (A) GRO⍺, (B) IL-1β, (C) IL-10, (D) MCP-1, (E) TNF⍺, and (F) IFNβ measurements of BMM∅s treated with SAHA-OH (30 µM), iNPs (300 µg/mL), iNP-SAHA_Low_ (300 µg/mL), or iNP-SAHA_High_ (300 µg/mL). SAHA-OH were pre-treated for 3 hours and subjected to LPS treatment for 48 hours. iNP, iNP-SAHA_Low_, and iNP-SAHA_High_ were incubated for 3 hours, excess NPs were washed with PBS, and subjected to LPS treatment for 48 hours. No treatment (NT) received no LPS stimulation, NP treatment, or SAHA-OH treatment. One-way ANOVA and Tukey’s multiple comparisons test was performed to determine statistical differences. *p<0.05, **p<0.01, ***p<0.001, and ****p<0.0001 compared to LPS control. All data is expressed as means ± SD (n=3).

**Figure S6. LPS mortality study analysis to establish lethality.** Dose escalation studies of i.p. administered 20 mg/kg, 30 mg/kg, or 40 mg/kg LPS (n=5 each). Kaplan-Meier curves and log-rank (Mantel-Cox) tests were performed to compare the survival rates to each survival group.
